# 2-hydroxyisobutyric acid (2-HIBA) modulates ageing and fat deposition in *Caenorhabditis elegans*

**DOI:** 10.3389/fmolb.2022.986022

**Published:** 2022-11-23

**Authors:** Emily Schifano, Giorgia Conta, Adele Preziosi, Carino Ferrante, Giovanni Batignani, Patrizia Mancini, Alberta Tomassini, Fabio Sciubba, Tullio Scopigno, Daniela Uccelletti, Alfredo Miccheli

**Affiliations:** ^1^ Department of Biology and Biotechnology “C. Darwin”, Sapienza University of Rome, Rome, Italy; ^2^ Department of Environmental Biology, Sapienza University of Rome, Rome, Italy; ^3^ NMR-based Metabolomics Laboratory of Sapienza (NMLab), Sapienza University of Rome, Rome, Italy; ^4^ Department of Physics, Sapienza University of Rome, Rome, Italy; ^5^ Center for Life Nano- and Neuro-science, Istituto Italiano di Tecnologia, Rome, Italy; ^6^ Department of Experimental Medicine, Sapienza University of Rome, Rome, Italy

**Keywords:** 2-hydroxyisobutyric acid, *Caenorhabditis elegans*, ageing, lipid metabolism, oxidative stress, high-glucose diet, metabolomics, CARS

## Abstract

High levels of 2-hydroxyisobutyric acid (2-HIBA) were found in urines of patients with obesity and hepatic steatosis, suggesting a potential involvement of this metabolite in clinical conditions. The gut microbial origin of 2-HIBA was hypothesized, however its actual origin and role in biological processes are still not clear. We investigated how treatment with 2-HIBA affected the physiology of the model organism *Caenorhabditis elegans*, in both standard and high-glucose diet (HGD) growth conditions, by targeted transcriptomic and metabolomic analyses, Coherent Anti-Stokes Raman Scattering (CARS) and two-photon fluorescence microscopy. In standard conditions, 2-HIBA resulted particularly effective to extend the lifespan, delay ageing processes and stimulate the oxidative stress resistance in wild type nematodes through the activation of insulin/IGF-1 signaling (IIS) and p38 MAPK pathways and, consequently, through a reduction of ROS levels. Moreover, variations of lipid accumulation observed in treated worms correlated with transcriptional levels of fatty acid synthesis genes and with the involvement of peptide transporter PEP-2. In HGD conditions, the effect of 2-HIBA on *C. elegans* resulted in a reduction of the lipid droplets deposition, accordingly with an increase of *acs-2* gene transcription, involved in β-oxidation processes. In addition, the pro-longevity effect appeared to be correlated to higher levels of tryptophan, which may play a role in restoring the decreased viability observed in the HGD untreated nematodes.

## 1 Introduction

The effects of complex factors resulting from the interactions between genetics and environment are known to be involved in the etiology of several multifactorial diseases like diabetes, cardiovascular diseases, cancer, *etc.* (Arneth et al., 2019; Mi et al., 2020; Amin, 2021; Guerra et al., 2021).

The metabolome of an organism consists of a huge variety of exogenous and endogenous low-molecular-weight molecules deriving from a large network of metabolic reactions (Wishart, 2019). It is of great interest to explore the role of specific metabolites or metabolic profiles in the diseases’ etiopathogenesis in order to gain insights about its development and progression and on the possible interventional practices.

The metabolite 2-hydroxyisobutyric acid (2-HIBA) was detected in mammalians’ urines. In particular it has been observed increasing in obese subjects, in alcohol consumers and in pregnant women who developed gestational diabetes mellitus, as well as women with healthy pregnancies (Calvani et al., 2010; Diaz et al., 2011; Elliott et al., 2015; Gil et al., 2018; Irwin et al., 2018). Moreover, in obese and in type 2 diabetic mice, higher urinary levels of 2-HIBA were observed (Li et al., 2017).

Several studies associated the presence of 2-HIBA with a reduced bacterial diversity in obese gut microbiota and, above all, with the presence of *Faecalibacterium prausnitzii*, frequently involved in dysbiosis, thus suggesting its contribution to the functions of the microbiota and intestinal health (Calvani et al., 2010; Miquel et al., 2014; Elliott et al., 2015; Yousri et al., 2015). However, the microbial origin of 2-HIBA in mammals is still not proven and, furthermore, its presence in humans’ and rodents’ stools samples has not been found (Preidis et al., 2014; Vernocchi et al., 2020; Conta et al., 2021; Ponziani et al., 2021; Marrocco et al., 2022).

On these bases, the dysregulation in glucose and lipid metabolism was hypothesized to be correlated to a higher content of urinary 2-HIBA, although its role in biological processes are still debated.

To study the molecular mechanisms of signal transduction pathways, the nematode *Caenorhabditis elegans* resulted to be a powerful animal model thanks to a completely sequenced genome and the 75% homology shared with that of mammals (Shen et al., 2018b; Markaki and Tavernarakis, 2020). It constitutes an excellent system that can be manipulated through an abundance of powerful cellular, molecular and genetic biology tools for the discovery of genes involved in various diseases by using mutant models (Shen et al., 2018a). Indeed, it allows tot study different cellular signalling, such as the oxidative stress and IIS pathways, which influence various human diseases, including diabetes (Zhou et al., 2019).

Furthermore, *C. elegans* has been used in studies of glucose-induced toxicity, demonstrating that high-glucose diet (HGD) affect growth, fertility, aging and lifespan (Alcántar-Fernández et al., 2019). HGD is known to generate reactive oxygen species (ROS), that include radical and non-radical oxygen species such as hydroxyl radical (HO.), superoxide anion (O_2_^−^), and hydrogen peroxide (H_2_O_2_), which can damage lipids, proteins and nucleic acids and could lead to cell death. In particular, the involvement of different pathways of lipid metabolism and oxidative stress such as SKN-1/NRF2, SBP-1/SREBP, and DAF-16/FOXO, has been previously observed (Alcántar-Fernández et al., 2018). To this end, worms can be used to study fat accumulation, through staining of lipid droplets (LDs), localized in gut granules and hypodermal cells (Vrablik et al., 2015).

The aim of this research was to investigate the effects of 2-HIBA supplementation on nematodes, in both wild type and mutants, on viability, ageing, motility, brood size, lipid droplets, oxidative stress, gene expression related to lipid metabolism and worms’ metabolic profile. We observed 2-HIBA effects on HGD condition as well. In order to deepen the involved molecular and cellular mechanisms underlying the responses mediated by the treatment with 2-HIBA, multiple approaches were applied, *i.e.*, fluorescence, real time PCR and NMR-based metabolomics.

## 2 Materials and methods

### 2.1 *C. elegans* strains and growth conditions

The *C. elegans* strains used were: wild-type N2, CL2166 (dvIs19 [(pAF15)gst-4p::GFP::NLS] III), CF1553 (*muls84[pAD76(Sod-3::GFP)*]), LD1 (ldIs7 [skn-1b/c::GFP + rol-6 (su1006)] and TJ356 (zIs356 [daf-16p::daf-16a/b::GFP + rol-6 (su1006)]) transgenic strains. Mutant strains used were KU25 *pmk-1* (km25), AU1 *sek-1* (ag-1), QV225 *skn-1* (zj15) and *pep-2* [*pept-1* (lg601)]. Nematodes were grown on nematode growth medium (NGM) and fed with heat killed *Escherichia coli* OP50 and 2-hydroxyisobutyric acid, 99% (Sigma-Aldrich, St. Louis, MO, United States) at different concentrations (5, 10 or 20 mM), as indicated. Afterward, 60 μl of heat-killed culture was spread on 3.5 cm diameter NGM plates and 60 μl of 2-HIBA dissolved in sterile H_2_O_dd_ was added. Heat-killed OP50 cells were prepared as follows: bacteria were cultured overnight in Luria-Bertani (LB) broth at 37°C, centrifuged at 6,000 rpm for 15 min and suspended in 2 ml of sterile water. Cells were then incubated at 65°C for 90 min and deposited onto NGM agar plates. Heat-killed cells were also plated on LB agar in parallel to ensure that no viable cells remained.

### 2.2 *C. elegans* lifespan assay

Synchronous nematodes were prepared as described in (Schifano et al., 2021), on NGM spread with *E. coli* OP50 and 2-HIBA at the concentrations of 0.1, 5, 10, 20, 50 or 100 mM . Nematodes grown on *E. coli* OP50 supplemented with 60 μl of sterile H_2_O_dd_ were taken as controls. Lifespan analysis was performed at 16°C and worms were daily transferred to new plates seeded with fresh lawns. They were scored as dead when they no longer responded to gentle touch with a platinum wire. At least 60 nematodes per condition were used in each experiment. To mimic high-glucose diet (HGD), 2% glucose (Sigma-Aldrich, St. Louis, MO, United States) was added to the mix of agar and salts of the NGM and experiments were performed at 20°C. All lifespan assays were performed in triplicate.

### 2.3 Brood size and body size analysis

Synchronized N2 worms were incubated at 16°C on NGM plates seeded with OP50 and 2-HIBA at the concentrations of 5, 10 or 20 mM, allowing embryos to lay. For fertility analysis, three individual animals were transferred onto a fresh plate every day, and the total number of progenies was counted with a Zeiss Axiovert 25 microscope. The procedure was repeated until the mother worms stopped laying eggs, at around day 6. Each day the progeny production was recorded, and we reported the sum from day 1 to day 6, which was finally compared with the untreated control. The experiment was performed three times.

For body length measures, animals were photographed from 1 to 5 days from egg hatching using a Leica MZ10F stereomicroscope connected to a Jenoptik CCD camera. Length of worm body was determined by using the Delta Sistemi IAS software and compared to untreated worms. At least 30 nematodes were analysed for each data set and at least three independent experiments were performed.

### 2.4 Pumping rate and lipofuscin analysis

About 10 worms of each condition were analyzed at the early stage (from day 1 to day 4) and during aging (from day 9 to day 11) of adulthood. Pumping rate was measured under Zeiss Axiovert 25 microscope by counting the number of grinder contractions of 10 animals for each treatment, during a period of 30 s, as described in (Guantario et al., 2018). To analyze the auto-fluorescence of lipofuscin granules, 10 worms per condition at different stages (day 2, 3, 5 and 11) were washed three times with M9 buffer and observed by Zeiss Axiovert 25 microscope under DAPI filter. Images were taken at the time of exposure of 0.2 s and scale bars were inserted by Zeiss ZEN Microscopy Software 2011. Fluorescence was analyzed using ImageJ software. Aging analyses were performed in triplicate.

### 2.5 Fluorescence analysis in the transgenic strains

At the stage of 1 day of adulthood, synchronized *gst-4*::GFP, *sod-3::GFP*, *daf-16*::GFP and *skn-1*::GFP transgenic worms fed heat killed OP50 and 10 mM 2-HIBA from embryo hatching were anesthetized with sodium azide (20 mmol L^−1^) (Sigma-Aldrich, St. Louis, MO, United States) and observed by Zeiss Axiovert 25 microscope as described in (Bianchi et al., 2020). The experiments were repeated three times and 15 worms per group were used in each experiment. Images were taken at the time of exposure of 0.2 s and fluorescence was analyzed using ImageJ software. Scale bars were inserted by Zeiss ZEN Microscopy Software 2011.

### 2.6 Evaluation of reactive oxygen species (ROS) levels

ROS formation in 1 day adult worms, treated or not with 10 mM 2-HIBA from embryo hatching, was measured using the fluorescent probe H_2_DCFDA, as described in (Yoon et al., 2018) with some modifications. Briefly, worms were collected (in triplicate) in a 96-well microplate and washed in the M9 buffer. H_2_DCFDA (Sigma-Aldrich, Milan, Italy) probe was added in each sample to obtain a final concentration of 50 μM. After 4 h of dark incubation at 20°C, worms were analyzed by using a microplate reader at excitation/emission wavelengths of 485 and 520 nm.

### 2.7 BODIPY staining

About 60 1 day adult nematodes, grown on heat-killed OP50 supplemented with 10 mM 2-HIBA (with or without glucose) from embryo hatching, were washed three times with M9 buffer. As described in (Schifano et al., 2019), worms were then incubated with a solution of 6.7 μg/ml BODIPY 493/503 (Thermo Fisher Scientific, Applied Biosystem, Invitrogen) for 20 min. Afterwards, nematodes were mounted onto 3% agarose pads containing 20 mM sodium azide and observed with Axio Observer Z1 inverted microscope, equipped with an ApoTome.2 System (Carl Zeiss Inc., Oberkochen, Germany). Fluorescence was analyzed using ImageJ software.

### 2.8 Coherent anti-stokes Raman scattering and two-photon fluorescence microscopy

A 7 picosecond laser source (Levante Emerald OPO, APE Angewandte Physik and Elektronik GmbH, Germany, pumped by a Nd:Vanadate laser at 1,064 nm, High Q Laser GmbH, Austria) generated two pulses at 76 MHz repetition rate, with powers of 70 mW and 120 mW for the pump (817 nm) and Stokes (1,064 nm) beams, that were spatially and temporally overlapped and then coupled to a modified inverted laser scanning microscope with a couple of galvo mirrors. A 40×/NA = 1.3 objective (Olympus UPLFLN40X) was used to focus both beams. This setup is able to generate CARS signals, when the focal spot is rich in CH vibration at 2,840 cm^−1^, as in the case of lipids. The emitted CARS signal (at 663 nm) and the two-photon fluorescence (in the range of 495–540 nm), collected through the same objective (EPI direction), were detected with two photomultipliers (Yen et al., 2010). One day adult worms, treated or not with 2-HIBA, were placed in a droplet of Levamisole (1 mM) on a 0.17-mm-thick microscopy cover glass covered with a thin agarose film. A second cover glass was then gently applied on the sample.

A 3D stacks of images was performed with a step of 2 μm. The images were collected over a square field of 150.5 μm with a later pixel size of 0.147 μm (1024 × 1024 pixels). For the calculation of lipid droplets, the slow modulations (not related to the lipid droplets) were removed subtracting the same image after a Gaussian blurring with standard deviation equal to 20 pixels. Then, the volume was calculated counting the pixels above a specific threshold (equal for each worm).

### 2.9 RT-qPCR

RNA of 200 1-day adults supplemented or not with 10 mM 2-HIBA from embryo hatching was extracted using miRNeasy Micro Kit (Qiagen) and real time analysis with I Cycler IQ Multicolor Real-Time Detection System (Biorad), was performed according to (Schifano et al., 2019). The selective primers (200 nM) for genes involved in fat accumulation and oxidative stress were reported in Supplementary Table S1. Quantification was performed using a comparative *C*_T_ method (*C*_T_ = threshold cycle value). Briefly, the differences between the mean *C*_T_ value of each sample and the *C*_T_ value of the housekeeping gene (*act-1*) were calculated: Δ*C*_Tsample_ = *C*_Tsample_—*C*_A*CT1*_. Result was determined as 2^−ΔΔCT^ where ΔΔ*C*_T_ = Δ*C*_Tsample_ − Δ*C*_Tcontrol_. The experiment was performed in triplicate.

### 2.10 Sample preparation and NMR analysis

For the metabolomics analysis, 7 couples of *C. elegans*, made of 7 samples of OP50 and 7 samples of nematodes treated with 10 mM 2-HIBA, were compared to 10 couples of *C. elegans* grown on a highly glucose-enriched diet, made of 10 samples of OP50 and 10 samples of nematodes treated with 10 mM 2-HIBA.

*C. elegans extraction procedure.* To about 3,000 frozen worms stored in polypropylene tubes, 1 ml of sterilized glass beads and 2 ml of iced MeOH (Merck KGaA, Darmstadt, Germany) were added. Nematodes were then subjected to 8 cycles of 2 min vortex and 2 min of rest in ice. The supernatant was picked up and the beads were washed with a mixture of 2 ml iced MeOH and 1 ml iced CHCl_3_ (Merck KGaA, Darmstadt, Germany), then vortexed. The supernatant was added to the previous one and the beads were again washed in 3 ml of iced CHCl_3_ and then vortexed. The final supernatant was added to the previous pool. Finally, 2 ml of H_2_O was added to the beads for washing, subsequently vortexed and the supernatant was added to the pool. Samples were kept overnight at 4°C. After the centrifugation at 10,000 × *g* at 4°C for 25 min, the hydroalcoholic and the chloroformic phases were separately collected, dried under N_2_ stream and preserved at −80°C until the subsequent analysis. Each dried polar sample was suspended in 700 μl of D_2_O containing 3-(trimethylsilyl)-propionic-2,2,3,3-D_4_ acid sodium salt (final concentration of TSP: 2 mM) (Sigma-Aldrich, St. Louis, MO, United States), as an internal chemical shift and concentration standard. The organic phase was instead resuspended in 700 μl of CDCl_3_ with hexamethyldisiloxane (final concentration of HMDS: 2 mM) (Sigma-Aldrich, St. Louis, MO, United States) as an internal standard.

*NMR analysis.* Five mm NMR glass tubes were used for the NMR analysis. All spectra were acquired at 298 K with 128 total scans, a spectral width of 9,025 Hz and 6,553 data points on a Jeol JNM-ECZ 600R spectrometer (JEOL Ltd., Akishima, Japan) operating at the proton frequency of 600 MHz, equipped with cryo-probe. Hydroalcoholic spectra were acquired employing the presat pulse sequence for solvent suppression (presat time = 2 s). The relaxation delay was set to 7.723 s to achieve complete resonance relaxation between following scansions.

To univocally identify the metabolites in the biological samples, bidimensional experiments ^1^H-^1^H Total Correlation Spectroscopy (TOCSY) and ^1^H-^13^C Heteronuclear Single Quantum Correlation (HSQC) were performed on selected samples.

TOCSY experiments (with DIPSY spin-lock) were conducted with a spectral width of 9,025 Hz in both dimensions, a data matrix of 8,192 × 256 points, a mixing time of 80 ms, and a relaxation delay of 2 s.

HSQC experiments were performed with spectral widths of 9,025 Hz and 37,764 Hz for the proton and carbon, respectively, a data matrix of 8,192 × 256 points and a recycle delay of 2 s.

Identification was confirmed by comparison with literature (Geier et al., 2011; Zanni et al., 2017), web database (Wishart et al., 2012) and in-house databases.

The spectra were processed by applying an exponential window function with a line broadening factor LB = 0.3 Hz; then, after the Fourier Transformation, they were manually phased and base corrected by applying the protocol BCFR. Metabolites’ quantification was carried out manually by comparing the integrals of specific metabolites resonances with the one of their specific internal standard and finally normalized for the number of protons. Once obtained the data expressed as concentration, a new datasheet that included both the polar and the non-polar metabolites for every group of samples (HGD, No-GD and treated with 2-HIBA and untreated worms) was created. All data were autoscaled before the multivariate and univariate analyses.

Monodimensional NMR spectra were processed and quantified by using ACD/Lab 1D NMR Manager ver. 12.0 software (Advanced Chemistry Development, Inc., Toronto, ON, Canada), whereas bidimensional NMR spectra were processed by using Delta NMR Software v 5.3.1 (JEOL Ltd., Akishima, Japan) and MestreNova v 11.0 (Mestrelab Research SL, Santiago de Compostela, Spain).

### 2.11 Statistical analysis

Multivariate analyses (PCA and PLS-DA) were performed with the Unscrambler ver. 10.5 software (Camo Software AS, Oslo, Norway) and univariate analyses were performed with SigmaPlot 14.0 software (Systat Software Inc., San Jose, CA, United States).

The statistical significance was performed by Student’s t-test or one-way ANOVA analysis coupled with a Bonferroni post-test (GraphPad Prism 5.0 software, GraphPad Software Inc., La Jolla, CA, United States). Differences with *p* values <0.05 were considered significant and were indicated as follows: *p < 0.05, **p < 0.01, and ***p < 0.001. Experiments were performed at least in triplicate. Data were presented as mean ± SD. For fluorescence images, mean fluorescence intensity was analyzed using the ImageJ software, measuring the ratio of pixels per area of the worm.

## 3 Results

### 3.1 Effects of 2-HIBA on wild-type worms physiology

To better clarify molecular mechanisms involved in 2-HIBA mediated cell responses, the viability rate was examined on wild-type nematodes, supplemented with concentrations of 2-HIBA equal to 0.1 mM, 5 mM, 10 mM, 20 mM, 50 mM, and 100 mM.

**FIGURE 1:**
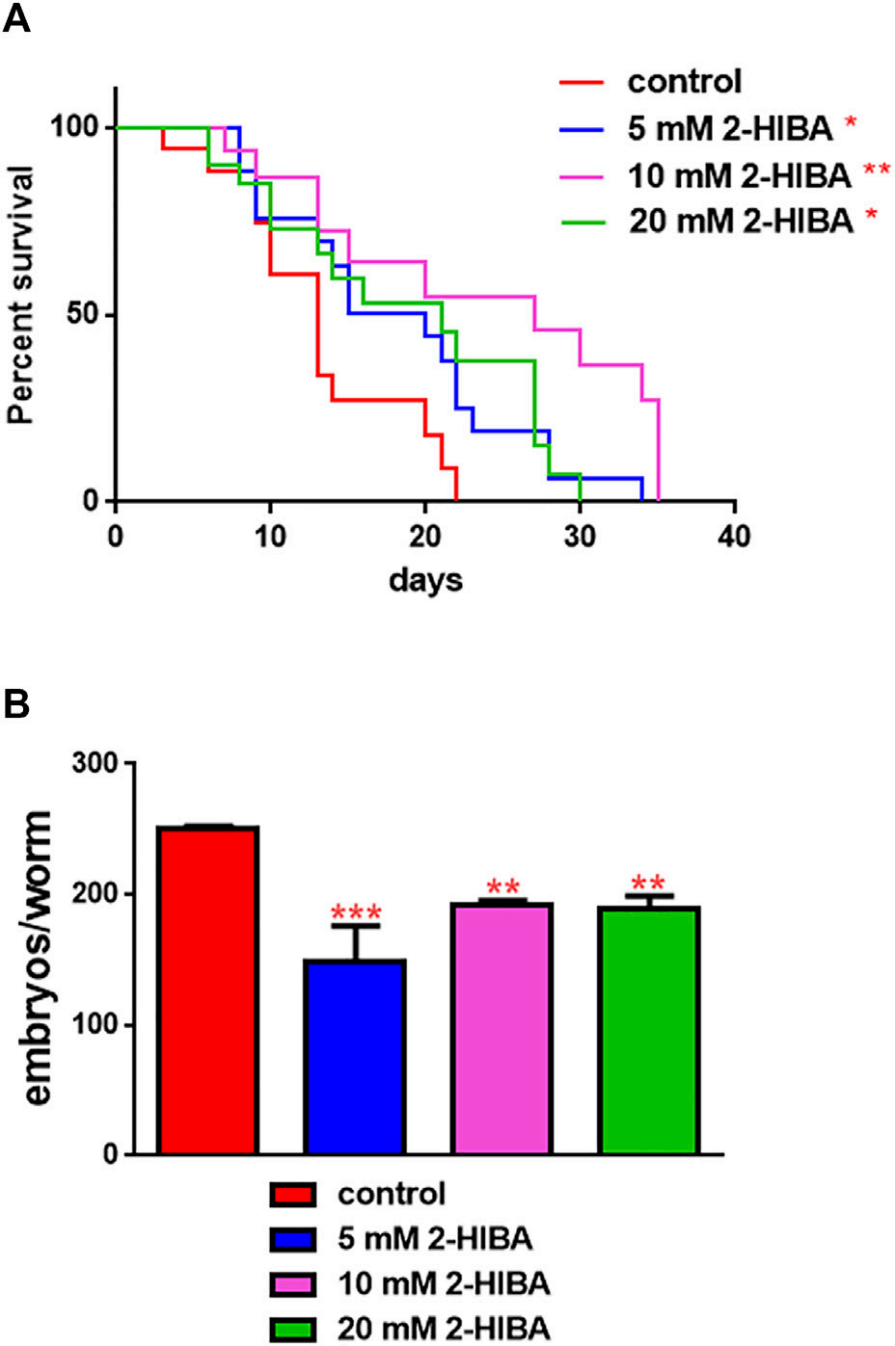
Impact of 2-HIBA on worm viability and fertility. **(A)** Kaplan–Mèier survival plot of N2 worms fed with heat-killed OP50 and supplemented with 5, 10 or 20 mM 2-HIBA. *n* = 60 for each data point of single experiments (*p < 0.05, **p < 0.01). Lifespan assay was performed in triplicate and OP50-fed worms were taken as reference. **(B)** Average embryos production per worm of animals supplemented with different concentrations of 2-HIBA Bars represent the mean of three independent experiments; asterisks indicate the *p*-values (log-rank test) normalized to the control (**p < 0.01, ***p < 0.001).

The median lifespan of wild-type worms fed with OP50 and supplemented with 10 mM 2-HIBA from embryo hatching was significantly extended as compared to the untreated controls (Figure 1A; Table 1). In particular, 50% of worm viability was recorded at day 25 in 10 mM treated worms, while in untreated animals it was recorded at day 12. The pro-longevity effect was also observed when nematodes were supplemented with 5 mM and 20 mM of 2-HIBA. Indeed, median survival in 5 mM- or 20 mM-supplemented worms was recorded at day 15 and day 20, respectively. On the other hand, 2-HIBA concentrations of 50 mM and 100 mM resulted in toxic effects for worms, while lower dosage of 0.1 mM did not exert a significant effect compared to untreated worms (Table 1).

**TABLE 1:** Lifespan analysis of wild-type N2, *sek-1, pmk-1, skn-1* mutant worms supplemented with 2-HIBA in standard or HGD condition. The lifespan of untreated animals was reported as control. Three experiments were performed in triplicate for each condition.

*C. elegans* strain	Diet	n	Median lifespan	Maximum lifespan	Number of censored	Statistics
Wild-type N2	Heat killed *E. coli* OP50	180	12 ± 0.2	20 ± 0.3	10	-
Wild-type N2	Heat killed *E. coli* OP50+ 0.1 mM 2-HIBA	180	13 ± 1.2	22 ± 1.1	12	ns versus N2 untreated control
Wild-type N2	Heat killed *E. coli* OP50 + 5 mM 2-HIBA	180	15 ± 0.5	34 ± 0.2	14	*p* < 0.05 versus untreated N2 control
Wild-type N2	Heat killed *E. coli* OP50 + 10 mM 2-HIBA	180	26 ± 0.08	35 ± 0.3	13	*p* < 0.01 versus untreated N2 control
Wild-type N2	Heat killed *E. coli* OP50 + 20 mM 2-HIBA	180	20 ± 1.4	30 ± 0.5	18	*p* < 0.05 versus N2 untreated control
Wild-type N2	Heat killed *E. coli* OP50 + 50 mM2-HIBA	180	10 ± 1.3	17 ± 1.3	12	*p* < 0.001 versus untreated N2 control
Wild-type N2	Heat killed *E. coli* OP50 + 100 mM 2-HIBA	180	8 ± 0.9	14 ± 0.09	14	*p* < 0.001 versus untreated N2 control
*sek-1* mutant	Heat killed *E. coli* OP50	180	13 ± 0.05	17 ± 0.5	10	-
*sek-1* mutant	Heat killed *E. coli* OP50 + 10 mM 2-HIBA	180	12 ± 1.3	16 ± 0.1	15	ns versus *sek-1* untreated control
*pmk-1* mutant	Heat killed *E. coli* OP50	180	13 ± 0.03	17 ± 0.9	15	-
*pmk-1* mutant	Heat killed *E. coli* OP50 + 10 mM 2-HIBA	180	12 ± 0.06	17 ± 0.05	17	ns versus *pmk-1* untreated control
*skn-1* mutant	Heat killed *E. coli* OP50	180	11 ± 0.1	15 ± 1.3	18	-
*skn-1* mutant	Heat killed *E. coli* OP50 + 10 mM 2-HIBA	180	11 ± 1.2	15 ± 0.7	12	ns versus *skn-1* untreated control
*pep-2* mutant	Heat killed *E. coli* OP50	180	13 ± 0.3	25 ± 0.08	19	-
*pep-2* mutant	Heat killed *E. coli* OP50 + 10 mM 2-HIBA	180	13 ± 0.9	25 ± 0.4	17	ns versus *pep-2* untreated control
Wild-type N2	HGD- Heat killed *E. coli* OP50	180	5 ± 0.8	11 ± 1.2	20	-
Wild-type N2	HGD-Heat killed *E. coli* OP50 + 5 mM 2-HIBA	180	10 ± 1.2	16 ± 0.8	18	*p* < 0.01 versus N2 untreated control
Wild-type N2	HGD- Heat killed *E. coli* OP50 + 10 mM 2-HIBA	180	7 ± 0.9	17 ± 0.4	15	*p* < 0.01 versus N2 untreated control
Wild-type N2	HGD- Heat killed *E. coli* OP50 + 20 mM 2-HIBA	180	7 ± 1.4	15 ± 1.3	19	*p* < 0.05 versus N2 untreated control

**FIGURE 2:**
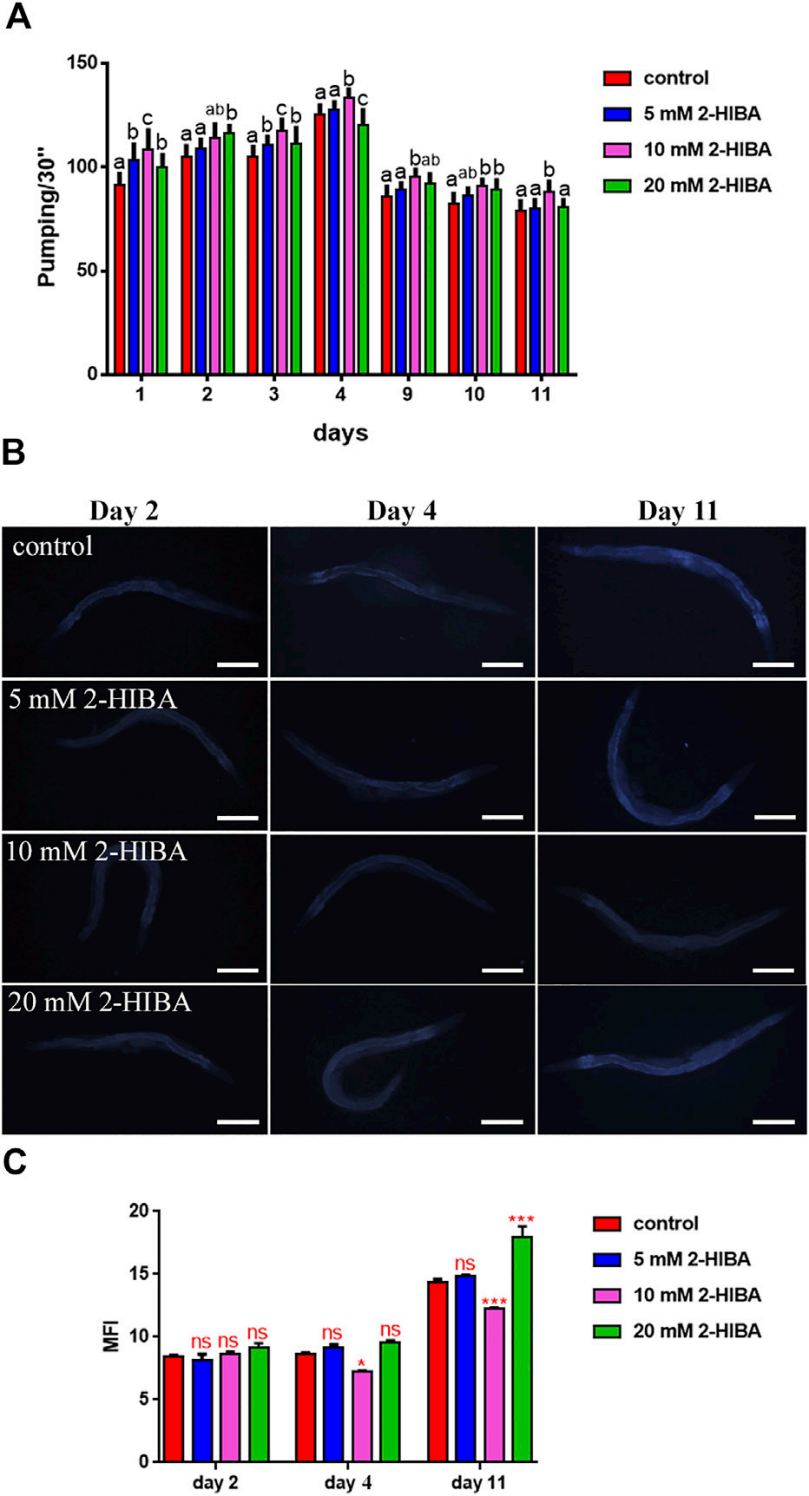
Effect of 2-HIBA on *C. elegans* ageing. **(A)** Pumping rate of worms at different stages after 2-HIBA treatment from embryo hatching and measured for 30 s. Pharyngeal contractions were determined from the mean of 10 worms for each bacterial strain. Worms fed heat-killed OP50 without 2-HIBA supplementation were used as controls (control: untreated worms). Different letters indicate statistically significant differences (*p* < 0.05). **(B)** Fluorescence microscopy of auto-fluorescent lipofuscin granules in *C. elegans* supplemented with 10 mM 2-HIBA. **(C)** Mean Fluorescence Intensity related to lipofuscin accumulation. Ten worms were used for each measurement (**p < 0.01). Scale bar = 100 μm.

Nematodes’ fertility was further analyzed evaluating the brood size, expressed as the number of embryos per worm, on worms treated with 5, 10 or 20 mM. A significant reduction of progeny production was observed in worms supplemented with all the three different concentrations of 2-HIBA*,* when compared to the control population (Figure 1B). In order to evaluate possible impact on larval development exerted by the molecule, the larval length was analyzed. Here we observed that the treatment with 2-HIBA did not show a particular effect on nematodes’ body length, except for a slight reduction in the size of treated animals at the fourth day from hatching (Supplementary Figure S1). However, this difference seemed to disappear in adulthood, suggesting that the impact of 2-HIBA, observed in *C. elegans* lifespan, did not depend on a delay on larval development. Ageing biomarkers were further analyzed in order to evaluate the effects of 2-HIBA on *C. elegans* ageing. To this aim, two ageing markers were considered: pumping rate and lipofuscin accumulation. By measuring contractions of the pharynx, we observed that nematodes supplemented with 10 mM 2-HIBA displayed an increased number of pharynx contractions with respect to the untreated nematodes, both during the stage of young adults and in old ones (Figure 2A). Moreover, data obtained from the quantification of lipofuscin accumulation showed that at day 11 of adulthood the auto-fluorescent pigment was reduced by about 20% in nematodes supplemented with 10 mM 2-HIBA (Figures 2B,C). These results showed that 10 mM 2-HIBA treatment had an impact in delaying ageing processes in *C. elegans*.

### 3.2 Oxidative stress in animals treated with 10 mM 2-HIBA

**FIGURE 3:**
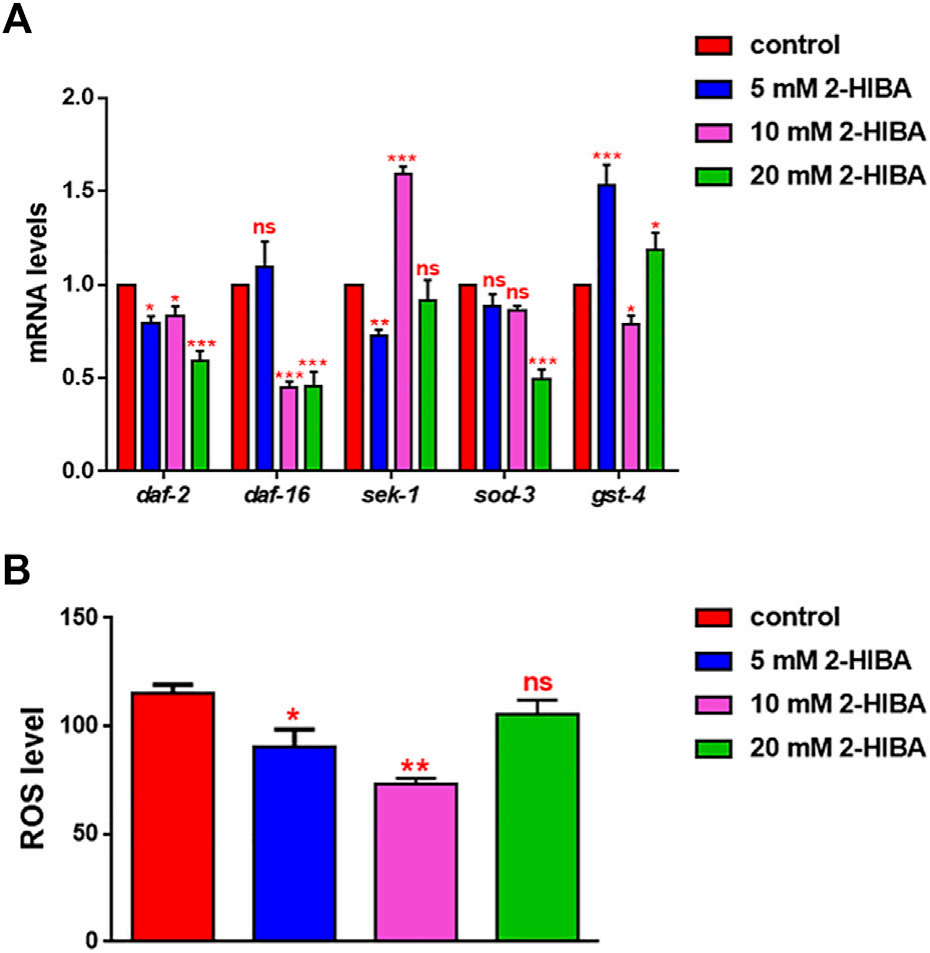
Impact of 2-HIBA on oxidative stress responses. **(A)** Expression of *daf-2*, *daf-16*, *sek-1*, *sod-3* and *gst-4* genes in N2 worms treated with 5, 10 or 20 mM 2-HIBA and in untreated nematodes at day 1 of adulthood. Histograms show the expression of genes involved in oxidative stress detected by real-time PCR. **(B)** Measurement of ROS levels in N2 supplemented with 2-HIBA compared to untreated control. Experiments were performed in triplicate. Data are presented as mean ± SD (*p < 0.05, **p < 0.01 and ***p < 0.001; ns: not significant).

**FIGURE 4:**
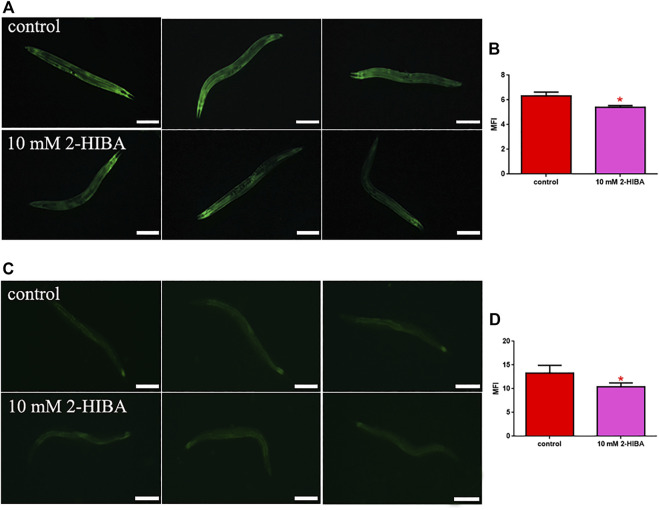
Fluorescence microscopy of *gst-4*::GFP and *sod-3*::GFP transgenic strains. **(A)** Fluorescence microscopy of *gst-4*::GFP worm strain after supplementation of 10 mM 2-HIBA and **(B)** related MFI. **(C)** Fluorescence microscopy of *sod-3*::GFP worm strain after supplementation of 10 mM 2-HIBA and **(D)** related MFI. Scale bar = 100 μm control: untreated worms. Statistical analysis was evaluated by one-way ANOVA with the Bonferroni post-test; asterisks indicate significant differences (*p < 0.05). Bars represent the mean of three independent experiments.

**FIGURE 5:**
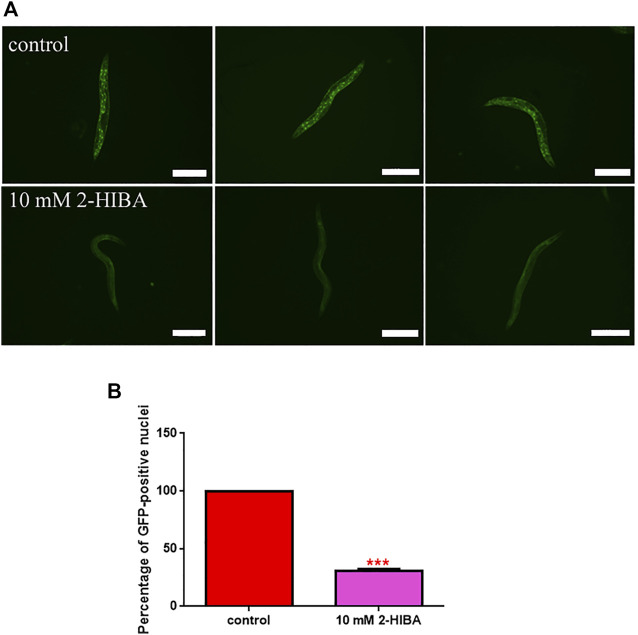
Fluorescence analysis of *daf-16*::GFP transgenic strain. **(A)** Effect of 10 mM 2-HIBA treatment on localization of DAF-16 protein and **(B)** respective Mean Fluorescence Intensity evaluation. Data were obtained from three independent experiments (60 worms for each condition). Scale bar = 100 μm control: untreated nematodes. Statistical analysis was performed by one-way ANOVA with the Bonferroni post-test; asterisks indicate significant differences (***p < 0.001). Bars represent the mean of three independent experiments.

**FIGURE 6:**
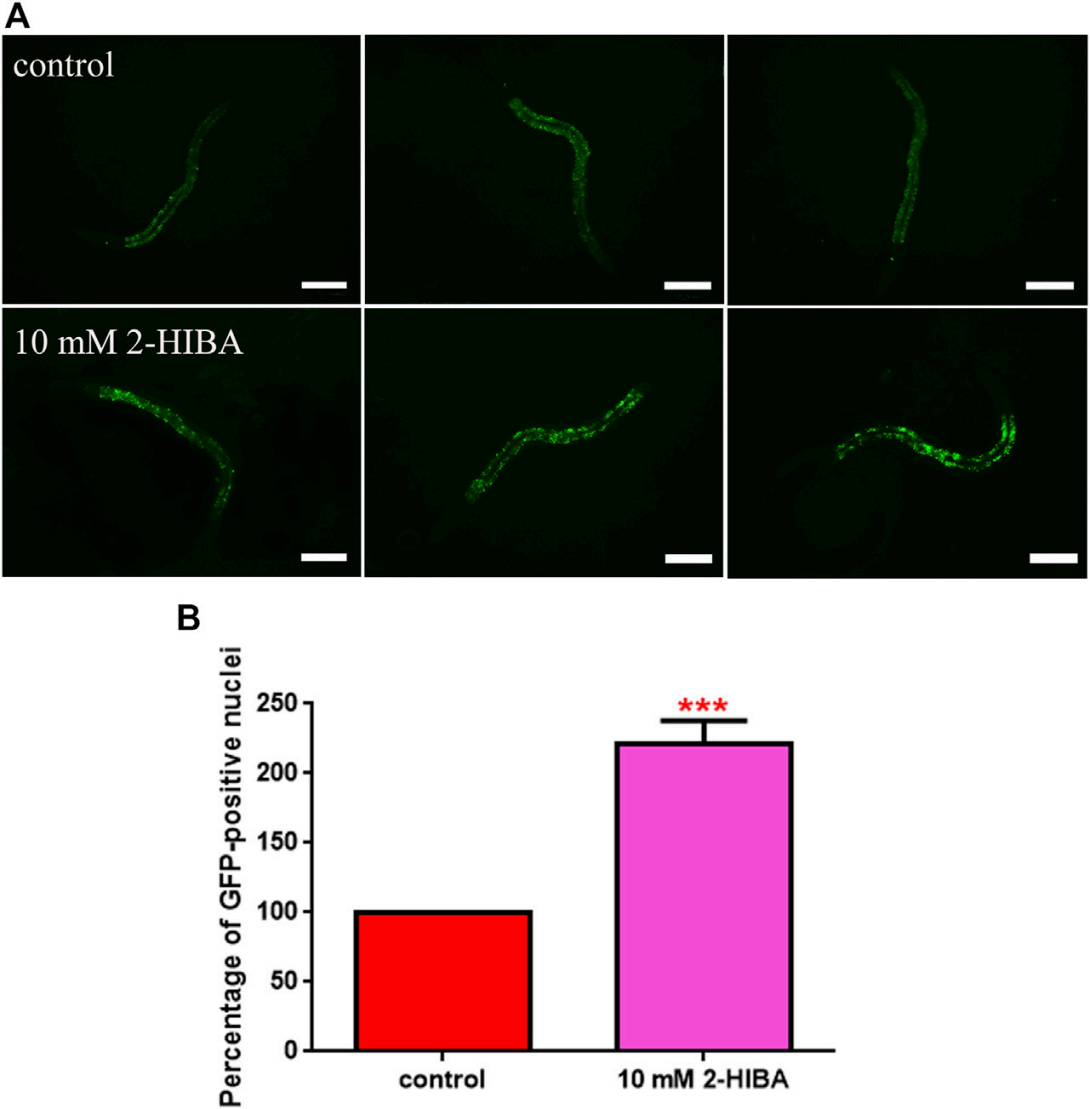
Fluorescence analysis of *skn-1*::GFP transgenic strain. **(A)** Effect of 10 mM 2-HIBA treatment on localization of SKN-1 transcriptional factor and **(B)** respective Mean Fluorescence Intensity evaluation. Scale bar = 100 μm. Data were obtained from three independent experiments (60 worms for each condition). control: untreated worms. Statistical analysis was performed by one-way ANOVA with the Bonferroni post-test; asterisks indicate significant differences (***p < 0.001). Bars represent the mean of three independent experiments.

**FIGURE 7:**
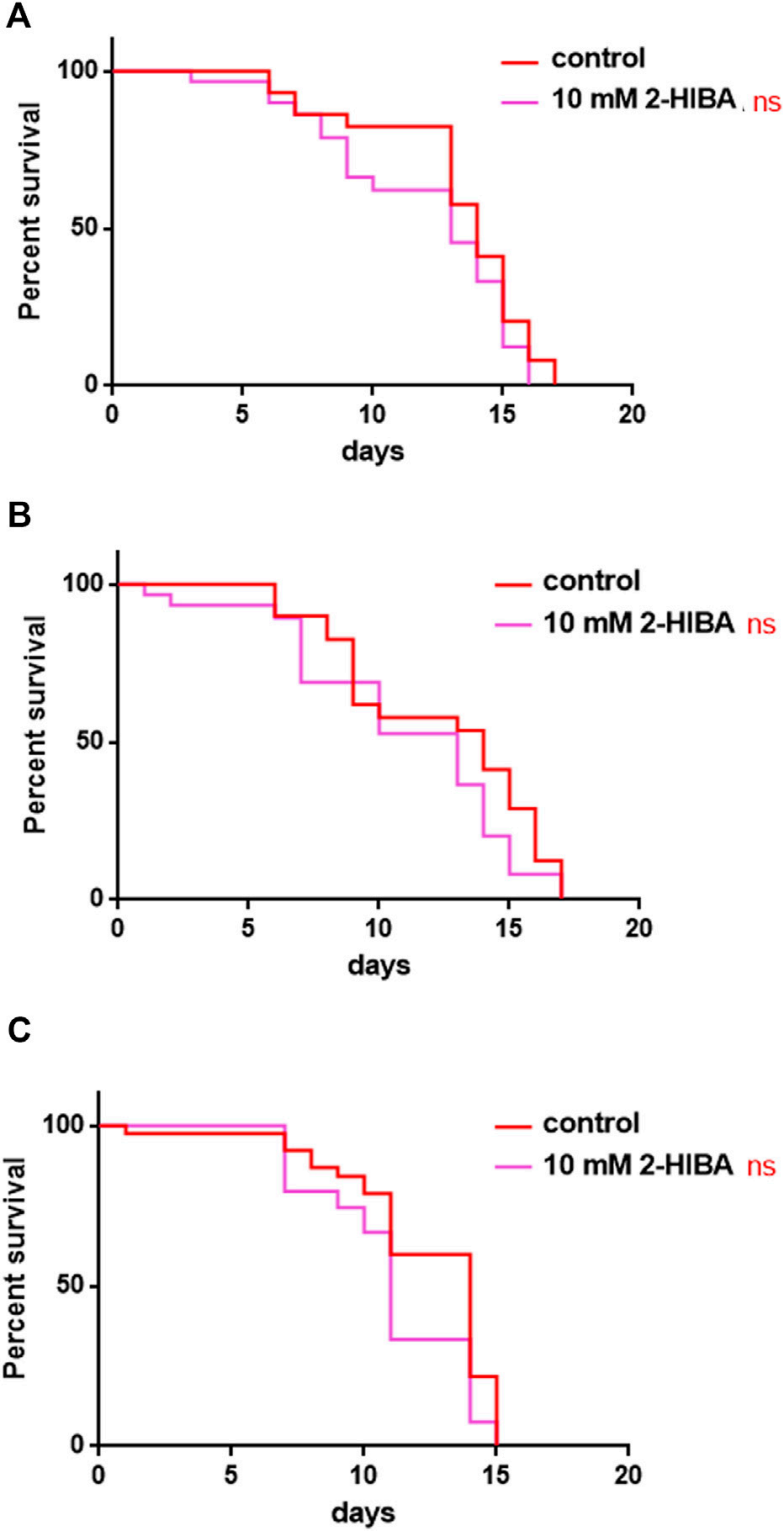
Effect of 10 mM 2-HIBA on *pmk-1*, *sek-1* and *skn-1* mutant animals. **(A)** Kaplan-Meier survival plot of **(A)** *pmk-1,* **(B)** *sek-1,* **(C)** *skn-1* mutant worms supplemented with 10 mM 2-HIBA. Lifespans of untreated worms (control) were taken as reference; *n* = 60 for each data point of single experiments (ns: not significant). The experiment was performed in triplicate.

During ageing, oxidative stress increases in *C. elegans*. Since pro-longevity and oxidative stress responses in nematodes are induced *via* the activation of different pathways, such as DAF-2/DAF-16 or MAPK cascades, a real time analysis was carried out, in order to understand possible mechanisms of action mediated by 2-HIBA. Transcripts analyzed include those encoding for the insulin-like growth factor 1 (IGF-1) receptor DAF-2, the forkhead box transcription factors class O (FoxO) homolog DAF-16, superoxide dismutase 3 (SOD-3) and glutathione s-transferase 4 detoxifying (GST-4) enzymes and the mitogen-activated protein kinase (MAPKK) SEK-1. In worms treated with 5 or 20 mM 2-HIBA, a general reduction in expression of genes involved in response to oxidative stress was observed, as compared to control (Figure 3A). In 10 mM 2-HIBA treated worms, an increased transcription of *sek-1* gene and a reduction of *daf-2, daf-16, sod-3* and *gst-4* was observed, with respect to the untreated worms. Accordingly, in worms supplemented with 10 mM 2-HIBA, ROS levels were 45% lower, as compared to control (Figure 3B). The results were confirmed by localization of corresponding protein in *gst-4*::GFP and *sod-3*::GFP transgenic strains (Figures 4A,C, respectively). In this case, the treatment with 2-HIBA induced a slight reduction of SOD-3 and GST-4 proteins, as confirmed by the quantification of fluorescence by Mean Fluorescence Intensity (MFI) (Figures 4B,C, respectively). Consistently, 10 mM 2-HIBA supplementation in *daf-16*::GFP and *skn-1*::GFP transgenic animals highlighted the involvement of p38 MAPK cascade and IIS pathway. Indeed, 1 day adult worms fed 2-HIBA 10 mM showed a reduced nuclear accumulation of DAF-16 by 70% (Figures 5A,B) as compared to the untreated nematodes. This phenomenon indicated that lifespan extension probably is DAF-16 independent. On the contrary, after the 2-HIBA administration to *skn-1*::GFP transgenic worms, a higher translocation of the p38 MAPK transcriptional factor SKN-1 was observed in nuclei, as compared to the control population. In particular, in treated nematodes the translocation resulted 2-fold higher than in the untreated ones (Figures 6A,B). Therefore, these results indicated that pro-longevity effects mediated by 2-HIBA could be SKN-1 dependent. To further support these results, the viability rate was further examined by administering 10 mM 2-HIBA to worms with mutations in *pmk-1*, *sek-1* and *skn-1* genes, encoding for proteins involved in p38 MAPK pathway. The median lifespan of *pmk-1* nematodes, supplemented with 10 mM 2-HIBA from embryo hatching, resulted similar to that of the untreated controls (Figure 7A; Table 1). In particular, 50% of worm viability in 10 mM 2-HIBA-supplemented nematodes was recorded at day 12, compared to controls in which, instead, it was recorded at day 13. A similar effect was also observed when the supplementation was added on *sek-1* mutant worms. Indeed, median survival in 10 mM 2-HIBA or untreated *sek-1* worms was recorded at day 12 and day 13, respectively (Figure 7B; Table 1). In the case of *skn-1* mutants, lifespan reached 50% of viability at day 11 in both conditions (Figure 7C; Table 1). Therefore, the molecule did not induce a pro-longevity effect in those mutants as observed in wild type animals, demonstrating that the effects mediated by 10 mM 2-HIBA involved the activation of p38 MAPK pathway.

### 3.3 Involvement of 2-HIBA in lipid and fatty acid metabolism

**FIGURE 8:**
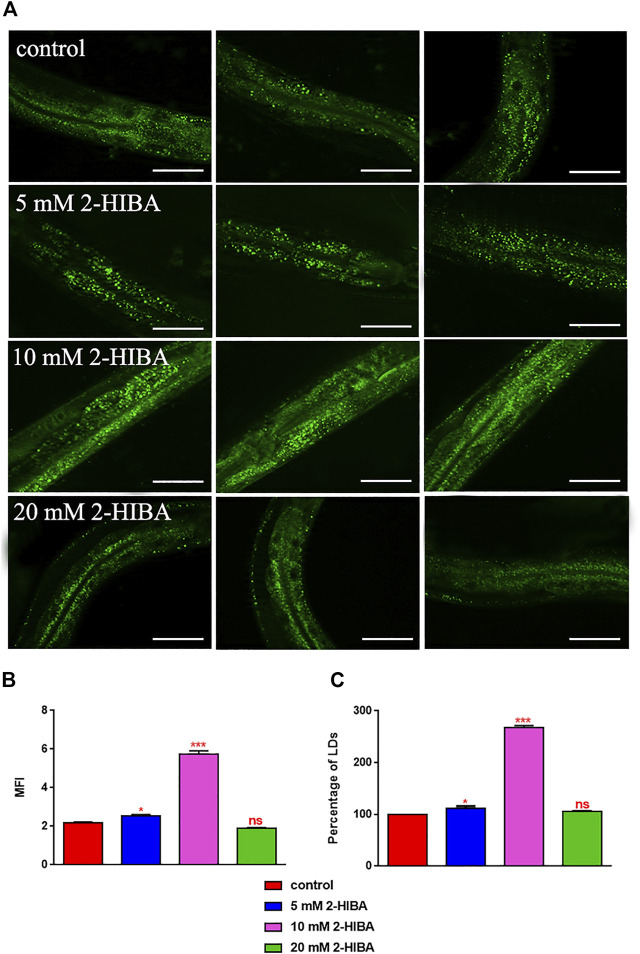
Visualization of lipid droplets. **(A)** BODIPY staining of 1 day adult worms treated or not with 5 mM, 10 mM or 20 mM 2-HIBA (control: untreated worms). Scale bar = 50 μm. **(B)** Related Mean Fluorescence Intensity and **(C)** percentage of lipid droplets. Statistical analysis was evaluated by one-way ANOVA with the Bonferroni post-test; asterisks indicate significant differences (*p < 0.05, ***p < 0.001, ns: not significant). Bars represent the mean of three independent experiments.

**FIGURE 9:**
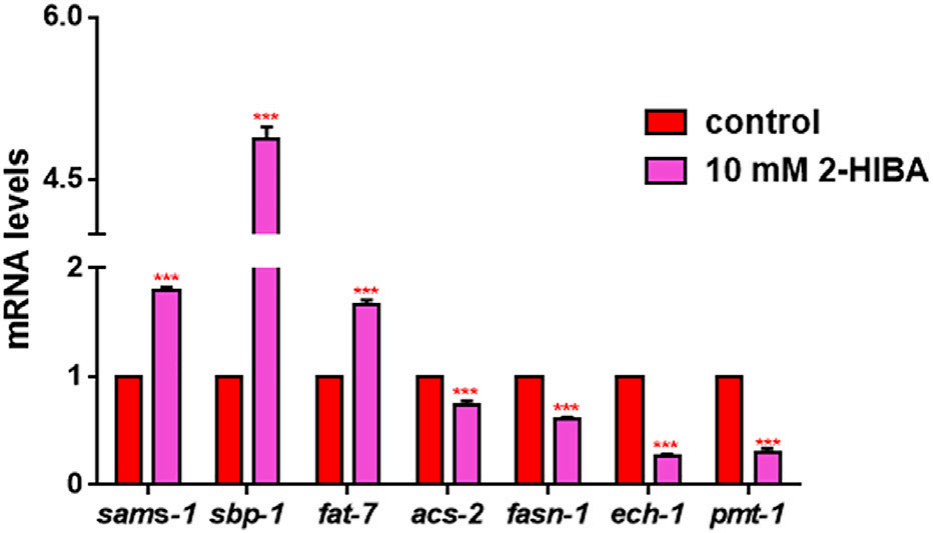
Real time-qPCR analysis of lipid metabolism genes in wild-type worms. Expression of genes involved in fat metabolism in 1 day adults treated or not with 10 mM 2-HIBA (control: untreated). Histograms show the expression of genes involved in lipid metabolism detected by real-time PCR. Experiments were performed in triplicate. Data are presented as mean ± SD (***p < 0.001).

Since ageing is related also to lipid metabolism, the effect of 2-HIBA was evaluated on the accumulation of *C. elegans* lipid reserves using the BODIPY fluorescent dye. The test was carried out on wild type *C. elegans* treated with 10 mM 2-HIBA and untreated (Figure 8). Images of fluorescence microscopy and lipid droplets quantification showed that animals supplemented with 10 mM 2-HIBA presented an increase in the accumulation of lipid droplets of about 60%, as compared to the control population. In particular, a more pronounced fluorescent signal highlighted an increase in lipid droplets both in size and in number with respect to the control. To identify the responsible genes for the differences in fat storage induced by 10 mM 2-HIBA, a real-time qPCR analysis was performed. The genes analyzed were S-AdenosylMethionine Synthetase (*sams-1*) and Phosphoethanolamine MethylTransferase (*pmt-1*), relevant for the synthesis of phosphatidylcholine (PC); the homolog of the mammalian transcription factor SREBP-1c (*sbp-1*), which facilitates fat storage in mammals; Delta9-fatty acid desaturase (*fat-7*) and Fatty Acid SyNthase (*fasn-1*), involved in the biosynthesis process of fatty acids; acyl-CoA synthetase (*acs-2*) and enoyl-CoA Hydratase (*ech-1*), involved in mitochondrial β-oxidation. The results showed that 2-HIBA induced an increased transcription of *sams-1*, *sbp-1* and *fat-7* genes related to the synthesis of lipids and a reduction of *acs-2* and *ech-1* genes, required for the mitochondrial β-oxidation, as compared to control (Figure 9). In particular, animals supplemented with 10 mM 2-HIBA presented an increased transcription of about 80% for *sbp-1*, 30% for *fat-7* and 50% for *sams-1* and a reduction of 20% for *acs-2* and 70% for *ech-1*, with the respect to control. The results showed an increasing effect of 2-HIBA on synthesis of fatty acids and a decreasing effect on degradation of fatty acids in wild type nematodes.

**FIGURE 10:**
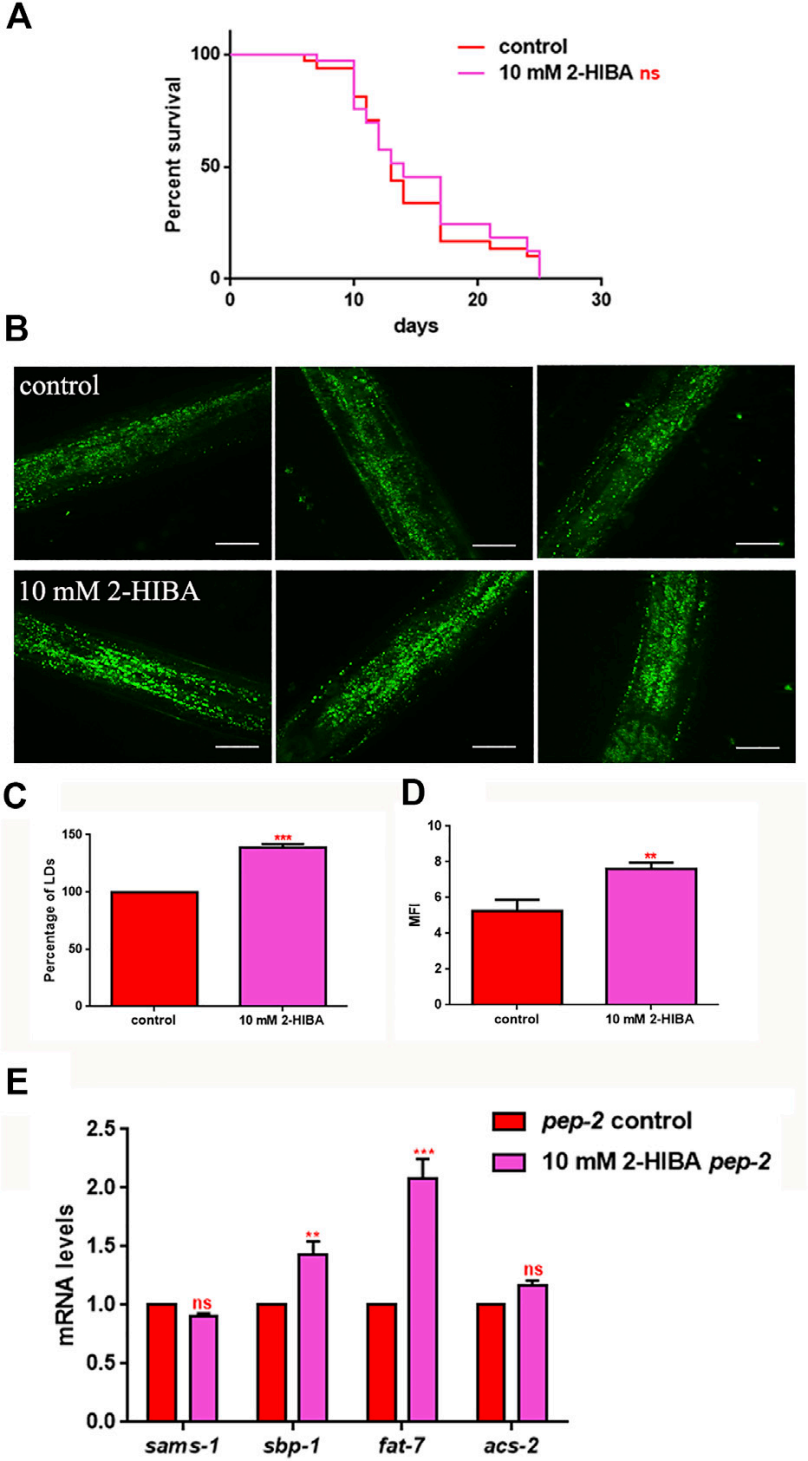
Lifespan analysis and fat accumulation in *pep-2* mutants. **(A)** Kaplan-Meier survival plot of *pep-2* mutant worms supplemented with 10 mM 2-HIBA. Lifespans of untreated worms were reported as controls; *n* = 60 for each data point of single experiments (ns: not significant). The assay was performed in triplicate. **(B)** BODIPY staining of 1 day adult *pep-2* worms treated or not with 10 mM 2-HIBA. Scale bar = 50 μm. **(C)** Percentage of Lipid Droplets (LDs) and **(D)** Mean Fluorescence Intensity (MFI) of fat accumulation observed by BODIPY staining. Statistical analysis was evaluated by one-way ANOVA with the Bonferroni post-test; asterisks indicate significant differences (**p < 0.01, ***p < 0.001). Bars represent the mean of three independent experiments. **(E)** Expression of *sams-1*, *sbp-1*, *fat-7*, and *acs-2* genes in 10 mM 2-HIBA-fed 1 day adults. Histograms show the transcript levels of genes involved in lipid metabolism in *pep-2* mutants, supplemented with 10 mM 2-HIBA, as compared to untreated control. Experiments were performed in triplicate. Data are presented as mean ± SD (***p < 0.001, ns: not significant).

It has been reported that one of the major genes involved in obesity is PEP-2, an intestinal proton-coupled peptide transporter (also known as PEPT-1 in mammals), which mediates amino acid absorption in the form of di- and tripeptides. Transporter-deficient animals (*pept-1(lg601)*) show impaired growth and metabolic alterations that culminate in a two-fold increase in total body fat content. The effects of 10 mM 2-HIBA on obese animals were therefore investigated. As shown in Figure 10A; Table 1, the treatment of 10 mM 2-HIBA in *pep-2* mutants led to an effect on lifespan similar to that observed on untreated nematodes. Indeed, the median survival of mutant worms was recorded at day 13 for both the conditions. Furthermore, fat accumulation in 10 mM 2-HIBA-fed *pep-2* mutants was about 50% higher than those detected in the untreated population (Figures 10B,C). To analyze fat metabolism gene expression in obesity model, a real time q-PCR was performed on *pep-2* mutants (Figure 10D). Nematodes showed a significant increase of *sbp-1*, *fat-7* and *acs-2* mRNA levels when treated with 2-HIBA, while variations in *sams-1* expression levels were not significant (Figure 10E). In particular, *pep-2* mutants supplemented with 10 mM 2-HIBA revealed an increased transcription of about 40% for *sbp-1*, 50% for *fat-7* and 20% for *acs-2*, as compared to untreated worms. Taken together, these results suggested the PEP-2 involvement in responses mediated by 2-HIBA.

### 3.4 Effect of 2-HIBA on *C. elegans* in high-glucose diet conditions

**FIGURE 11:**
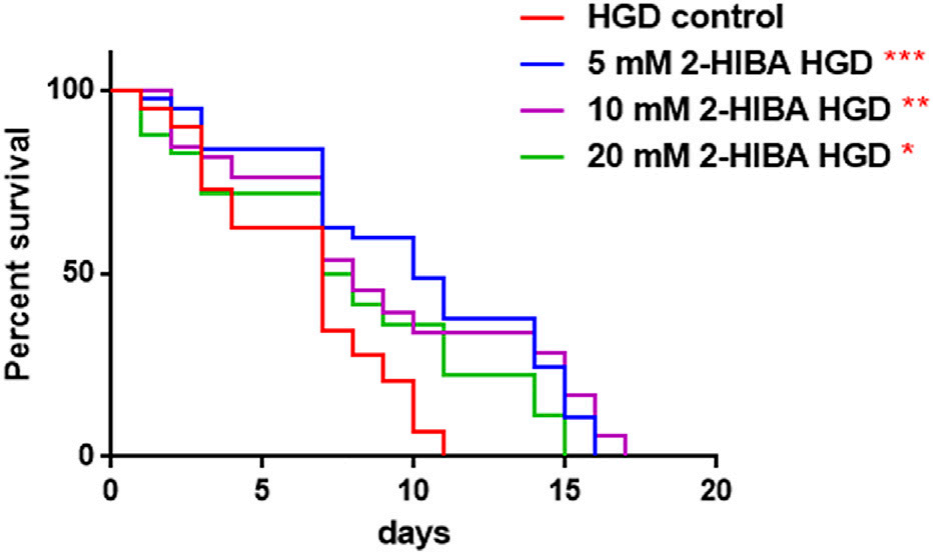
Effect of 2-HIBA on HGD wild-type worms (A) Kaplan–Mèier survival plot of HGD worms treated or not with 2-HIBA at different concentrations (control: untreated). *n* = 60 for each data point of single experiments. Bars represent the mean of three independent experiments. Asterisks indicate the *p*-values (log-rank test) with respect to the untreated control (*p < 0.05, **p < 0.01, ***p < 0.001).

**FIGURE 12:**
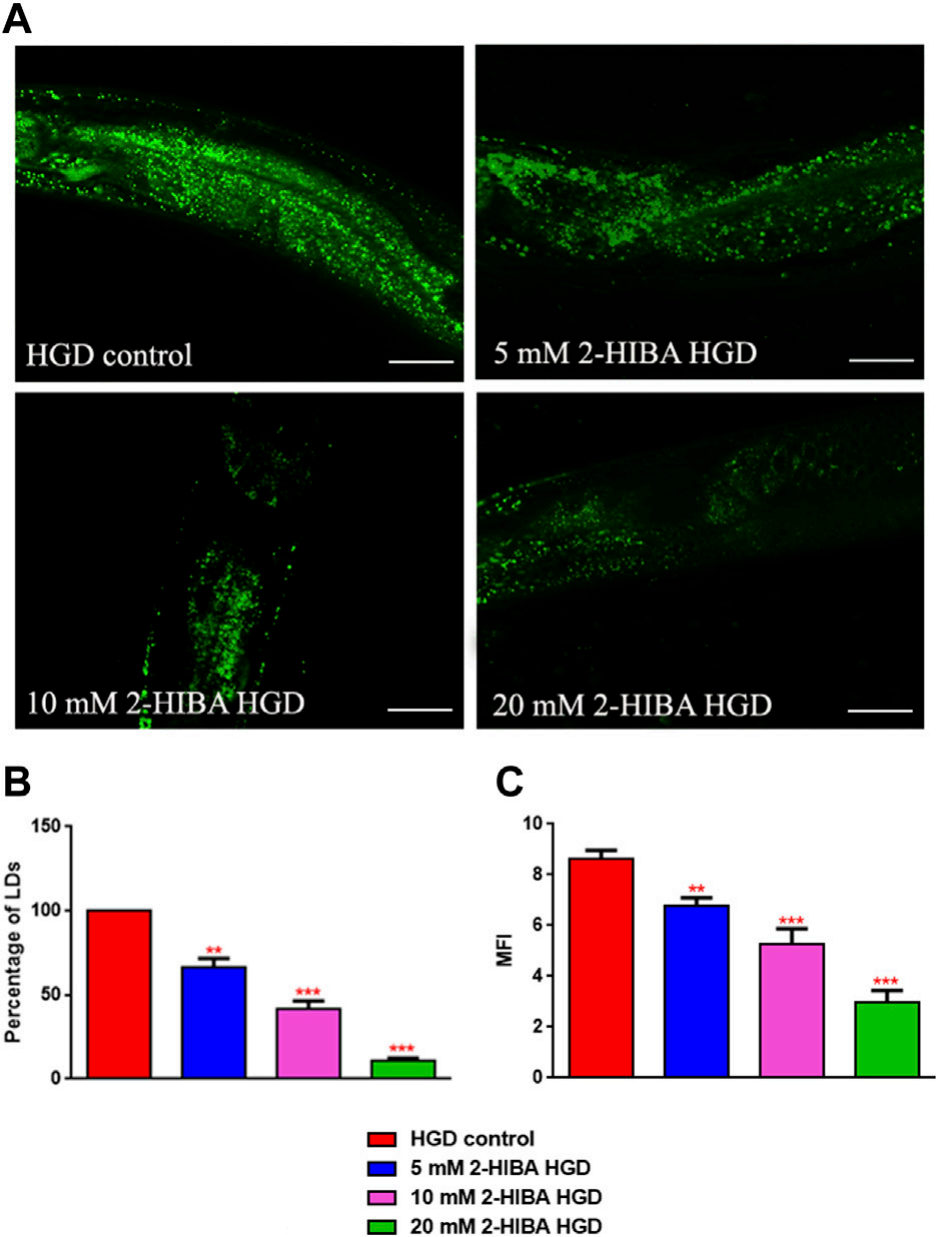
Lipid droplets accumulate in HGD worms. **(A)** BODIPY staining and **(B,C)** lipid droplets quantification in 1 day adult worms grown in the presence of glucose and fed with *E. coli* OP50 alone (control) or supplemented with different 2-HIBA concentrations (**p < 0.01, ***p < 0.001). Scale bar = 50 μm.

**FIGURE 13:**
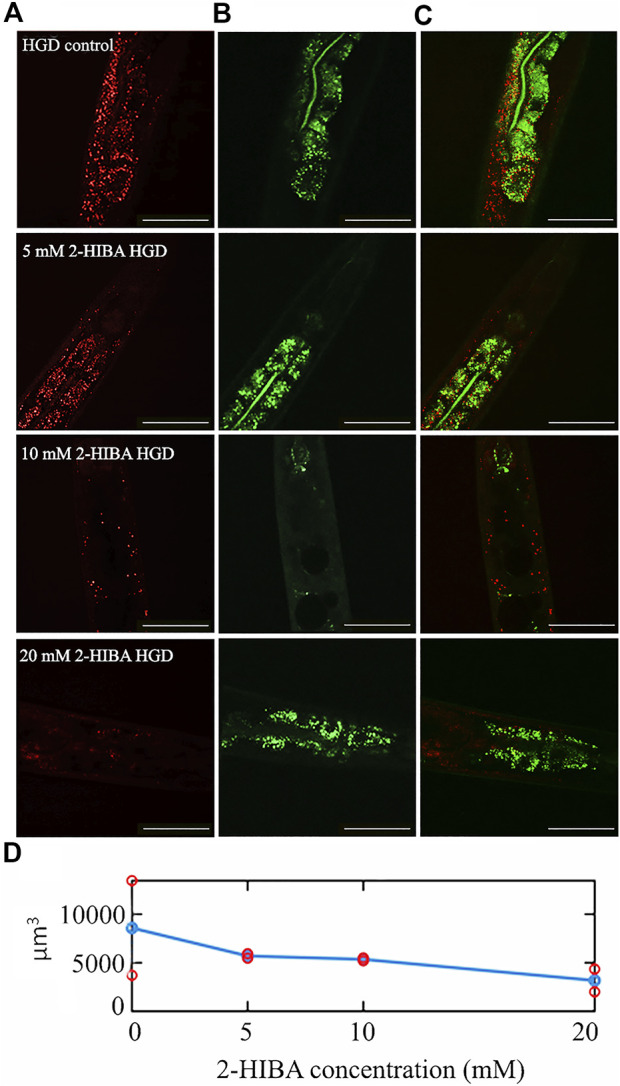
CARS microscopy of HGD worms treated with 2-HIBA at different concentrations. **(A)** CARS signals of lipid droplets and **(B)** two-photon auto-fluorescence image in early intestine and hypodermal cells in 1 day adult HGD worms treated or not with different concentrations of 2-HIBA, collected in the same region though our two channel system. The combined image is reported in **(C)**. The scale bar = 50 µm. **(D)** The lipid volume, detected in the CARS 3D stacks of images (two for each concentration), was reported by red circles, while the average was shown with blue circles.

**FIGURE 14:**
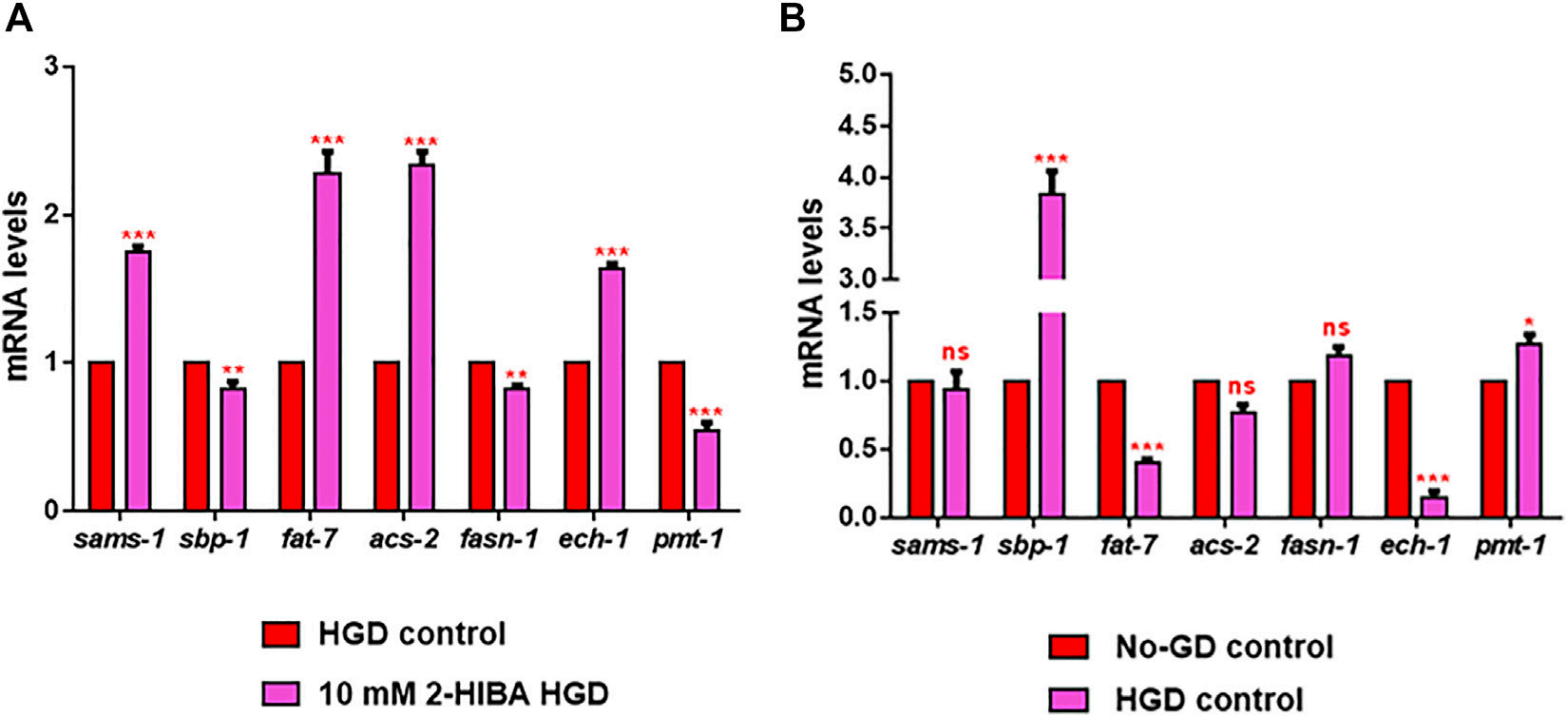
RT-qPCR analysis of lipid metabolism genes in HGD worms. **(A)** Expression of *sams-1*, *sbp-1*, *fat-7*, *acs-2, fasn-1, ech-1* and *pmt-1* genes in 2-HIBA HGD 1-day adults, compared to HGD untreated nematodes. **(B)** Expression of lipid metabolism genes in untreated populations, grown with (HGD) or without (No-GD) glucose. Experiments were performed in triplicate. Data are presented as mean ± SD (*p < 0.05, **p < 0.01, ***p < 0.001, ns: not significant).

Moreover, it has been reported that high-glucose diet (HGD) also affects growth, fertility, ageing and lifespan, through the activation of the IIS pathway. Thus, it was investigated whether 2-HIBA was able to counteract glucose toxicity by testing different concentrations of 2-HIBA in wild type animals exposed to 2% glucose. Untreated worms showed a decrease of viability in HGD conditions and median survival at day 5 (Figure 11; Table 1). On the contrary, 2-HIBA supplementation partially restored the worm’s lifespan. In particular, in worms treated with 5 mM 2-HIBA, the 50% of viability was reached at day 10. In worms treated with 10 mM or 20 mM of 2-HIBA was recorded a median viability at day 7. Since HGD also influences fatty acid metabolism, the effect of 2-HIBA on the accumulation of lipid reserves in *C. elegans* was evaluated by using the BODIPY fluorescent dye. In the presence of glucose, BODIPY staining detected large amounts of intestinal fat in untreated worms. Notably, 2-HIBA administration reduced that fat amount in a dose-dependent manner. In particular, histograms representing the percentage of lipid droplets highlighted a reduction of 30%, 50% and about 80% in worms treated with 5, 10 and 20 mM, respectively, as compared to control (Figure 12). Results were confirmed by Coherent Anti-Stokes Raman Scattering (CARS) analysis (Figure 13). CARS microscopy consists in a label-free chemical imaging technique, allowing direct visualization of lipid-rich organelles due to the abundance of the CH_2_ group. In agreement with BODIPY visualization, CARS images and fluorescence quantification showed that animals grown with HGD and supplemented with 2-HIBA revealed a proportional decrease in the accumulation of lipid droplets, as compared to the untreated population. Notably, among different concentrations, 10 mM 2-HIBA was able to significantly reduce lipofuscin auto-fluorescence, as highlighted by two photon fluorescence images (Figure 13). Based on this set of results, also subsequent experiments were performed using 10 mM 2-HIBA. Interestingly, in HGD worms treated with 2-HIBA was observed a higher expression of *acs-2* and *ech-1* (involved in β-oxidation), although there was an increase in the expression of genes involved in lipid synthesis (Figure 14A). In particular, animals supplemented with 10 mM 2-HIBA presented an increased transcription of about 70% for *sams-1* and *ech-1* and 1.5-fold increase for *fat-7* and *acs-2* genes, with the respect to the untreated control, while variations in *sbp-1* expression were not significant. Notably, comparing untreated nematodes grown with or without glucose, in HGD worms a significant 2.5-fold increase in *sbp-1* expression and a 50% reduction in *fat-7* and *ech-1* transcript levels were observed (Figure 14B).

### 3.5 NMR-based metabolomics

By ^1^H-NMR metabolomics, a total of 43 metabolites, belonging to the classes of amino acids, fatty acids (FA), short chain fatty acids (SCFA), organic acids, glycerols, nitrogen compounds, nucleosides, carbohydrates, were identified and quantified (Supplementary Table S2). Comparing all the categories analyzed, only quantitative, but no qualitative differences were observed. Representative hydroalcoholic and chloroformic ^1^H-NMR profiles are shown in the supplementary material (Supplementary Figures S2, S3).

As the first step, it was performed a Principal Component Analysis (PCA) on the entire dataset composed of 14 samples of nematodes put on a high-glucose diet (HGD), of which 7 were untreated and 7 were treated with 10 mM 2-HIBA, and 14 samples grown without glucose (No-GD), 7 treated with 10 mM 2-HIBA and 7 untreated was performed. The scores plot showed that the HGD samples were separated from the No-GD ones along the PC2 (Supplementary Figure S4).

Furthermore, the PLS-DA was performed on the controls matrix showed R^2^ = 0.98 and Q^2^ = 0.79 for the discrimination of nematodes grown on a glucose substrate (HGD) from those who grew without it (No-GD). The HGD group showed higher levels of betaine, glycine (Gly), adenosine-X-phosphate (AXP) and lower levels of monoacylglycerols (MAG), N-acetyl- moieties, lactate, tyrosine (Tyr), tryptophan (Trp), formate and U03 than No-GD. Univariate analysis, performed on each of these significant variables confirmed the statistical significance of MAG, Gly, Trp, formate and AXP between the HGD and No-GD groups.

In order to observe the effect of HGD on 2-HIBA treated worms, a further PLS-DA analysis was performed on the fold-ratio matrix (paired 2-HIBA/untreated samples) on a reduced space of variables. The classification model showed R^2^ = 0.86 and Q^2^ = 0.51 and higher levels of Trp and acetate in the 2-HIBA-treated HGD group and were also statistically significant at the univariate analysis. The HGD group treated with 2-HIBA showed a 2.5 fold change as compared to the untreated.

## 4 Discussion

### 4.1 The effects of 2-HIBA on *C. elegans* grown in standard conditions

Among the different concentrations tested on *C. elegans*, 10 mM 2-HIBA was found particularly effective to extend the lifespan, to delay ageing processes and to stimulate the oxidative stress responses in wild type nematodes. Microscopy analysis demonstrated that 2-HIBA did not affect larval development but it reduced fertility, possibly because nematodes spent more energy to survive and to contrast the ageing processes rather than to produce progeny, consistently with a previous work (Lee et al., 2015). As compared to the untreated worms, the pumping rate in treated old worms was higher and lipofuscin granules were reduced. Indeed, pumping rate consists in the number of contractions of the nematode’s grinder, normally decreasing with ageing, while auto-fluorescence granules of lipofuscin accumulate along the old worm intestinal tissues (Papaevgeniou et al., 2017). These results suggested a pro-longevity effect of 2-HIBA linked to a delay of ageing processes in *C. elegans*.

Moreover, during ageing, Reactive Oxygen Species (ROS) are normally accumulated in organisms, generating an impairment correlateing with cellular damages and a diminished lifespan (Miranda-Vizuete and Veal, 2017). Interestingly, in 2-HIBA treated worms we observed the activation of both p38 MAPK and IIS pathways and, therefore, a reduction of ROS levels.

The involvement of p38 MAPK pathways has been confirmed by the viability analysis performed on mutant animals depleted in genes encoding respectively for SEK-1, PMK-1 and SKN-1 proteins, all belonging to the same MAPK signaling. The lifespan extension observed in wild-type worms was completely absent in these mutants supplemented with 2-HIBA. Migration of transcriptional factor SKN-1 into the nucleus was further corroborated by fluorescence microscopy.

The activation of the IIS pathway, that prevented DAF-16 from entering the nucleus and avoiding downstream *sod-3* and *gst-4* genes transcription, was confirmed by real-time q-PCR and fluorescence analysis. These observations confirmed the stimulation of 2-HIBA on both p38 MAPK and IIS pathways activation. Altogether, these results demonstrated that the effect of 2-HIBA on nematodes maintained the organisms’ health and counteracted the adverse effects of ageing, through mechanisms of resistance to oxidative stress. Our data were consistent with previous studies that correlated pro-longevity and anti-ageing effects with a reduction of cellular stress (Miranda-Vizuete and Veal, 2017; Schifano et al., 2019). The increase of lipid droplet accumulation in 2-HIBA treated animals correlated with the activation of IIS pathway (CIT) and the decrease in the expression of *acs-2* gene, coding for a key crucial enzyme involved in the fatty acids β-oxidation pathway (Coleman et al., 2002). These data, along with the enhanced expression of *sams-1*, *sbp-1* and *fat-7* genes involved in lipid biosynthesis, strengthen the evidence that the accumulation of fat storage in treated nematodes was probably due to an imbalance between synthesis and degradation of lipids. Among others, of particular interest was *fat-7* gene, which has been reported in maintaining appropriate levels of saturated and unsaturated fats in response to nutritional intake, inhibiting fatty acid β-oxidation and *acs-2* expression (Mullaney et al., 2010).

To evaluate the role of 2-HIBA in obese worms, *pep-2* mutants were exploited. They lack intestinal di- and tri-peptide transporter that is involved in worm development and growth (Schifano et al., 2019). Nematode mutants show an increase of body fat, resistance to oxidative stress, and a reduction in body size and progeny compared to N2 worms (Meissner et al., 2004; Spanier et al., 2009). Unlike wild type N2 strain, 10 mM 2-HIBA administration was not able to prolong life and to increase *sams-1* transcripts in *pep-2* worms. Amino acid uptake through PEP-2 activation is regulated by the DAF-2/DAF-16 pathway (Baumeister et al., 2006). Moreover, regarding lipid accumulation, the slight increase of *acs-2* transcript levels, although significant, did not reach the high values of those of *fat-7* and *sbp-1* synthesis genes, emphasizing an additive effect of 2-HIBA on obese worms. These results were strongly linked to the increase of lipid accumulation observed with BODIPY staining.

### 4.2 The effects of 2-HIBA on *C. elegans* supplemented with a high-glucose diet

Glucose is an essential energy source for many cellular processes. The high-glucose diet (HGD) is related to obesity and pathological conditions, and it is known to be linked to a higher production of ROS. Indeed, an altered glucose homeostasis negatively influenced the development, fertility and lifespan of organisms as yeasts, worms, and mammals (Schulz et al., 2007; Edwards et al., 2015; Lee et al., 2015; Alcántar-Fernández et al., 2018). The increase in triglycerols (TAG) levels were associated to the expansion and the hydrolysis inhibition of lipid droplets (Li et al., 2016). Consistently, in our study we observed an increase of TAG and a decrease of monoacylglycerols (MAG) levels in HGD worms compared to No-GD worms, which is also correlated with the BODIPY staining. Normally, TAG stored in cytoplasmic lipid droplets were hydrolyzed by cytosolic enzymes in lipolytic products such as diacylglycerols, monoacylglycerols, glycerol, fatty acids. They serve as energy resources, for lipid remodelling, membrane biosynthesis, and signalling molecules (Hofer et al., 2020). Lipid profile in terms of saturated and unsaturated fatty did not differ between HGD and No-GD, as well as between 2-HIBA treated and untreated worms. On the contrary, MAG, TAG and phospholipids content showed a variation between HGD and No-GD, however the treatment with 2-HIBA appeared not to have an influence on those metabolites.

Our results also showed that reduced viability in HGD N2 worms was partially restored by 2-HIBA treatment, even if the median lifespan values did not reach the ones for worms grown in standard condition (No-GD). The metabolomics analysis highlighted a significant increase in tryptophan (Trp) levels in treated worms supplemented with HGD. Previous studies reported that higher levels of this amino acid were associated with lifespan extension in nematodes (van der Goot and Nollen, 2013; Edwards et al., 2015). Specifically, in *C. elegans* lifespan extension is known occurring in dietary restriction conditions (Lee et al., 2006). It was also observed along with the depletion of tryptophan 2,3-dioxygenase (TDO), an enzyme able to metabolize free Trp in the kynurenine pathway of tryptophan degradation (van der Goot and Nollen, 2013), suggesting a down-regulated activity of degradation of tryptophan by 2-HIBA treatment. Pro-longevity effects in worms also depend on SKN-1 signaling (Edwards et al., 2015) and on the transcription factor DAF-16, which act in the IIS pathway (Zečić and Braeckman, 2020), therefore, the role of TDO might converge on some of these longevity factors. The previous data suggested that *daf-16* could be involved in lifespan regulation by *tdo-2* (van der Goot et al., 2012). Unlike the No-GD conditions, microscopy analyses on HGD nematodes treated with different concentrations of 2-HIBA revealed a dose-dependent decrease of intestinal and hypodermal lipid droplets, in terms of distribution and morphology. Treatment with 10 mM 2-HIBA on HGD worms showed a further increment in the expression of *sams-1* and *fat-7* genes. However, in these nematodes also *acs-2* levels increased, unlike the reduction observed in No-GD treated animals. This could explain an enhancement in the β-oxidation process, compared to the untreated ones, resulting in a reduction in lipid vesicles.

Our data showed that the 2-HIBA-dependent inverse relationship between *fat-7* and *acs-2* genes could be only observed in wild type nematodes in No-GD conditions, similarly to what was previously reported in conditions of starvation (Nomura et al., 2010). Conversely, in HGD conditions we observed a decrease of both *acs-2* and *fat-7* genes. Moreover, it caused a significant increase of the transcription of *sbp-1*. The caloric excess stimulates *sbp-1* expression in *C. elegans* same as for *SREBP-1c* in mammals.

Unexpectedly, for both obese *pep-2* model and HGD worms, we observed the increase in *acs-2* and *fat-7* genes, depending on 2-HIBA treatment. *fat-7* and *acs-2* expression is regulated by NHR-49 in response to short fasting through its interactions with SBP-1 (Nomura et al., 2010). NHR-49 is necessary for the expression of genes involved in fatty-acids-β-oxidation and lipid binding, while SBP-1 appears to be involved in the expression of genes that participate in fatty acid synthesis. A possible relation between 2-HIBA and NHR-49 needs to be investigated.

## 5 Conclusion

Overall, 2-HIBA treatment extended lifespan and induced anti-ageing effects, protecting against oxidative stress in *C. elegans*. These responses, in standard conditions, seem to occur through induction of insulin/IGF-1 signaling (IIS) and p38 MAPK pathways and are dependent on PEP-2 activation. In HGD conditions, the pro-longevity effect appeared to be correlated to higher levels of Trp, which might play a role in restoring the decreased viability observed in the HGD untreated nematodes. The effect of 2-HIBA on HGD worms resulted in a reduction of the lipid droplets deposition, accordingly with an increase of acs-2 and ech-1 genes transcription, involved in β-oxidation processes.

Based on what we observed in HGD condition, we inferred that the presence of 2-HIBA in human urines could be linked to counteracting the accumulation of lipids as a response to a diet characterized by a high carbohydrate content. The origin of 2-HIBA and its role in the HGD-dependent Trp degradation mediated by TDO pathway will deserve further investigations.

## Data Availability

The original contributions presented in the study are included in the article/Supplementary Materials, further inquiries can be directed to the corresponding author.
