# Supplementary material for: 2-hydroxyisobutyric acid (2-HIBA) modulates ageing and fat deposition in Caenorhabditis elegans
Source: Front Mol Biosci. 2022 Nov 23;9:986022. doi: 10.3389/fmolb.2022.986022 (PMC9749906; doi:10.3389/fmolb.2022.986022)

Supplementary Material

Table S1. Primers for real time qPCR analysis.

act-1	FOR	5′-GAGCGTGGTTACTCTTTCA
	REV	5′-CAGAGCTTCTCCTTGATGTC
sek-1	FOR	5′-CAGAGCCGTTTATTGGGAAA
	REV	5′-TGCATCCGGCTTGTACAGT
sod-3	FOR	5′-AGAACCTTCAAAGGAGCTGATG
	REV	5′-CCGCAATAGTGATGTCAGAAAG
gst-4	FOR	5′-TCAATGTGCCTTACGAGGATTA
	REV	5′-CGAATTGTTCTCCATCGACTTG
daf-2	FOR	5′-ATCGTCGGATTCTACTGTACTCCC
	REV	5′-CCGACATCTGACAATATTCATTCTCGT
daf-16	FOR	5’-TCAAGACCTCAAAGCCAATCAACTC
	REV	5’-ACGAGAAAGAAGGAGTAAGAGGAGG
sams-1	FOR	5′-GAGAAGATGTCGGAGCAGGAGA
	REV	5′-TGTGGGAAAGAATGAGAGTCAATGG
sbp-1	FOR	5′-GGATGATATTGCGCCATTTC
	REV	5′- GGATCCGGTTGTTGTGATG
	FOR	5′-TGGCGGTAACGTGGCTCTCTTTG

fat-7	REV	5′-TAGGTCGAGTTTGCCTCCATGC
acs-2	FOR	5′-AGTGAGACTTGACAGTTCCG
	REV	5′-CTTGTAAGAGAGGAATGGCTC
fasn-1	FOR	5’- TGCTTCTTGCTGGTGAAGAC-3′
	REV	5’- GTATCTGGGTCTTGTGTGA-3′
ech-1	FOR	5’- GATATAGCCTCTGTATTGG-3′
	REV	5’-AATTGTCCTCCACTCTTCGC-3′
pmt-1	FOR	5′-ACTGCTCGCTGGGTCTTGTC-3′
	REV	5′-TCTCCGACTTGATAGTTGATGTTCCC-3′

Table S2 a. List of metabolites assigned and quantified in the aqueous and organic extracts of C. *elegans*. The assignment was performed on the basis of 2D-NMR TOCSY and HSQC experiments. In bold are highlighted the resonances chosen for the metabolite quantification. s: singlet, bs: broad singlet, d: doublet, t: triplet, dd: doublet of doublets, m: multiplet. A: aqueous fraction metabolites; O: organic fraction metabolites.

	**Compound**	**Group**	**^{1}H (ppm)**	**Molteplicity**	**Note**
1.	Val	**CH_3**	**0,99**	d	A
		CH_3	1,05	d	
		CH	2,27	m	
		α-CH	3,61	d	
2.	Ile	CH_3	0,94	t	A
		**CH_3**	**1,01**	d	
			1,28	m	
			1,48	t	
			1,97	m	
			3,67	d	
3.	Propionate	**CH_3**	**1,06**	t	A
		CH_2	2,19	m	
4.	3-hydroxyisobutyrate (3-HIB)	**CH_3**	**1,08**	d	A
			2,52		
			3,55		
			3,72		
5.	3- hydroxybutyrate (3-HB)	**CH_3**	**1,19**	d	A
		CH_2	2,40		
		CH	4,16		
6.	3-hydroxyisovalerate (3-HIV)	**CH_3, CH_3'**	**1,26**	s	A
		CH_2	2,35	s	
7.	Lactate (LA)	**CH_3**	**1,33**	d	A
		CH	4,11	m	
8.	Ala	**CH3**	**1,48**	d	A
		α-CH	3,79	m	
9.	Lys	ϒ-CH_2	1,46		A
		**δ-CH_2**	**1,72**	m	
			1,94		
		ε-CH_2	3,04		
		α-CH	3,76		
10.	Acetate (AA)	**CH_3**	**1,92**	s	A
11.	Acetamide	**CH_3**	**1,98**	s	A
12.	N-acetyl-	**CH_3**	**2,02**	s	A
13.	5-aminopentanoic ac		1,68		A
			1,90		
		**CH_2**	**2,25**	t	
			3,68		
14.	Glu	β-CH	2,06		A

		β-CH’<br>**ϒ-CH_2**<br>α-CH	2,14<br>**2,36**<br>3,76	 m 	
15.	Succinate (SA)	**α,β-CH_2**	**2,41**	s	A
16.	Gln	β-CH_2<br>**ϒ-CH_2**<br>α-CH	2,13<br>**2,46**<br>3,78	m<br>m 	A
17.	Phosphocholine	**3 CH_3**	3,21	s	A
18.	Choline	**3 CH_3**<br>CH_2<br>CH_2	**3,22**<br>3,50<br>4,05	s<br>m<br>m	A
19.	Betaine	**3 CH_3**<br>CH_2	**3,27**<br>**3,89**	s<br>s	A
20.	Gly	**CH_2**	**3,56**	s	A
21.	Glucose	 <br><br>**α-CH**<br>**β-CH**	3,25<br>3,57<br>3,71<br>**4,65**<br>**5,23**	 <br><br>d<br>d	A
22.	Trehalose	 <br><br>**CH, CH**	3,56<br>3,65<br>3,78<br>**5,20**	 <br><br>d	A
23.	U01		**5,39**	s	A
24.	U02		3,80<br>4,14<br>**5,86**	 m<br>d	A
25.	Fumarate	**CH, CH’**	**6,52**	s	A
26.	Tyr	**CH-1, CH-5**<br>CH-2, CH-4	**6,90**<br>7,20	d<br>d	A
27.	His	CH-2 ring<br>CH-5 ring	7,09<br>7,85	bs<br>bs	A
28.	Phe	**5 CH ring**	**7,33-7,45**	m	A
29.	Trp	CH<br>**CH**	7,20<br>**7,49**	 d	A
30.	Formate	**CH**	**8,46**	s	A
31.	U03		**7,68**	s	A
32.	U04		**8,44**	s	
33.	Nicotinamide riboside	**CH**	**8,95**	bs	A
34.	NAD	 <br><br><br>**CH**	8,18<br>8,44<br>8,84<br>9,16<br>**9,35**	s<br>s<br>d<br>d<br>s	A
35.	CXP	**CH**	**5,96-5,99**		A

		CH	6,12-6,15	d	
36.	UXP	**CH**<br>CH	**5,90-5,97**<br>7,89-7,90	 d	A
37.	GXP	**CH**	**8,09-8,17**	s	A
38.	AXP	**CH**	**8,58**	s	A
39.	Saturated fatty acid	CH_3<br>n-CH_2<br>CH_2-CH_2-COOH<br>**CH_2-COOH**	0.87<br>1.26<br>1.62<br>**2.30**	t<br>m<br>m<br>t	O
40.	Polyunsaturated FA ω-3	CH_3<br>n-CH_2<br>CH_2-CH=CH<br>CH=CH<br>**=CH-CH_2-CH=**<br>CH_2-CH_2-COOH<br>CH_2-COOH	0.95<br>1.37<br>2.04<br>5.36<br>**2.82**<br>2.03<br>2.30	t<br>m<br>m<br>m<br>t<br>m<br>t	O
41.	Polyunsaturated FA ω-6	CH_3<br>n-CH_2<br>CH_2-CH=CH<br>CH=CH<br>**=CH-CH_2-CH=**<br>CH_2-CH_2-COOH<br>CH_2-COOH	0.86<br>1.36<br>2.04<br>5.37<br>**2.76**<br>2.06<br>2.31	t<br>m<br>m<br>m<br>t<br>m<br>t	O
42.	Monoacylglycerols (MAG)	**CH_2**<br>CH_2<br>CH_2	**3,66**<br>4,15<br>3,90	dd<br>dd<br>m	O
43.	Triacylglicerols (TAG)	**2CH_2**	**4,25**	dd	O
44.	Phospholipids	**CH**	**5,23**	bs	O

Table S2 b. Metabolites mean and standard deviation. Data expressed as nmol/n, n= 1000 worms.

**Metabolite**	**No-GD**		**HGD**	
	**CTRL**	**2-HIBA**	**CTRL**	**2-HIBA**
**Saturated fat**	561.3±171.28	540.74±201.43	687.26±179.99	712.73±302.52
**w-6**	381.39±187.89	388.62±213.55	409.48±98.31	419.85±103.12
**w-3**	157.88±67.99	158.43±82.70	145.5±28.33	151.13±31.51
**Monoacylglycerol**	196.06±22.87	183.81±21.92	111.58±28.99	120.83±14.32
**Triglycerides**	124.49±57.42	124.57±61.30	191.23±63.23	194.82±84.64
**Phospholipids**	112.33±51.36	112.74±57.44	165.24±37.73	171.1±37.18
**Val**	29.30±21.16	31.34±21.81	29.55±8.36	31.46±11.49
**Ile**	18.59±14.38	18.75±14.39	20.92±8.20	23.57±9.61
**Propionate**	33.74±32.55	31.97±33.47	10.8±4.51	11.87±4.33

**3-HIB**	2.55±2.07	2.68±2.25	1.13±0.37	1.28±0.66
**3-OH-but**	6.29±4.14	5.98±4.83	3.99±2.45	4.35±2.61
**3-OH-isoval**	0.56±0.38	0.58±0.38	0.51±0.65	0.46±0.67
**Ala**	303.55±173.31	301.92±174.15	371.3±134.91	394.46±152.62
**Lys**	64.44±51.57	65.89±54.58	68.02±18.63	71.19±24.67
**Acetate**	75.52±27.64	68.53±25.27	58.85±10.61	66.4±12.98
**Acetamide**	19.00±11.74	20.56±11.69	22.68±6.65	23.78±6.53
**N-acetyl-**	9.17±6.80	9.00±7.50	3.71±1.07	3.70±0.95
**5-aminopentanoic ac**	25.75±15.76	24.35±16.56	41.41±28.62	46.52±33.44
**Glu**	83.06±48.73	88.15±56.64	89.69±36.66	93.24±34.76
**Succinate**	32.78±23.58	33.59±24.18	60.74±51.27	60.35±51.32
**Gln**	21.12±12.18	20.26±12.18	37.86±15.1	40.29±16.24
**Phosphocholine**	8.75±8.78	9.12±10.15	11.76±4.89	13.29±6.15
**Choline**	9.74±4.54	9.84±4.06	12.06±4.98	12.48±4.58
**Betaine**	6.33±4.68	6.55±5.71	8.18±4.19	8.05±3.11
**Gly**	34.45±26.45	34.96±26.25	160.56±62.22	176±79.75
**Lactate**	92.63±41.47	85.22±37.89	63.73±42.73	64.15±40.44
**Glc**	31.76±19.83	32.79±21.05	27.13±15.01	30.76±17.75
**Threalose**	32.67±16.92	33.15±17.08	60.34±52.59	70.06±66.3
**U01**	2.44±1.09	2.43±1.01	2.42±1.42	2.78±1.8
**U02**	2.60±0.90	2.64±0.76	4.90±2.67	5.31±3.61
**CXP+UXP**	23.25±16.77	22.99±16.07	25.06±6.34	27.35±8.34
**Fumarate**	0.46±0.47	0.39±0.42	2.29±1.16	2.30±0.85
**His**	15.16±8.82	14.23±9.24	14.26±4.4	16.15±7.02
**Tyr**	13.06±8.54	12.74±8.78	11.92±3.81	12.29±4.51
**Phe**	19.58±15.01	18.78±15	16.46±4.4	17.11±4.83
**Trp**	3.89±1.48	3.12±0.75	0.59±0.32	1.20±0.49
**U03**	8.99±6.98	5.96±4.44	5.84±1.79	6.54±4.30
**GXP**	8.20±7.43	8.01±7.55	10.17±2.88	11.14±2.04
**U04**	1.72±0.69	1.88±0.75	1.67±0.7	1.79±0.65
**Formate**	80.77±31.22	77.54±31.26	7.72±0.71	8.19±1.00
**AXP**	17.53±13.05	19.46±9.37	35.79±11.51	37.65±10.63
**NamRib**	2.66±2.06	2.78±1.99	1.79±0.89	2.16±1.56
**NAD**	2.32±0.77	2.25±0.82	2.79±1.10	2.82±1.06

Figure S1. Analysis of body length in worms fed heat-killed OP50 supplemented with different concentrations of 2-HIBA. Length of worms during larval development was measured from head to tail at the indicated time points. control: untreated worms. Different letters indicate statistically significant differences ($p < 0.05$).

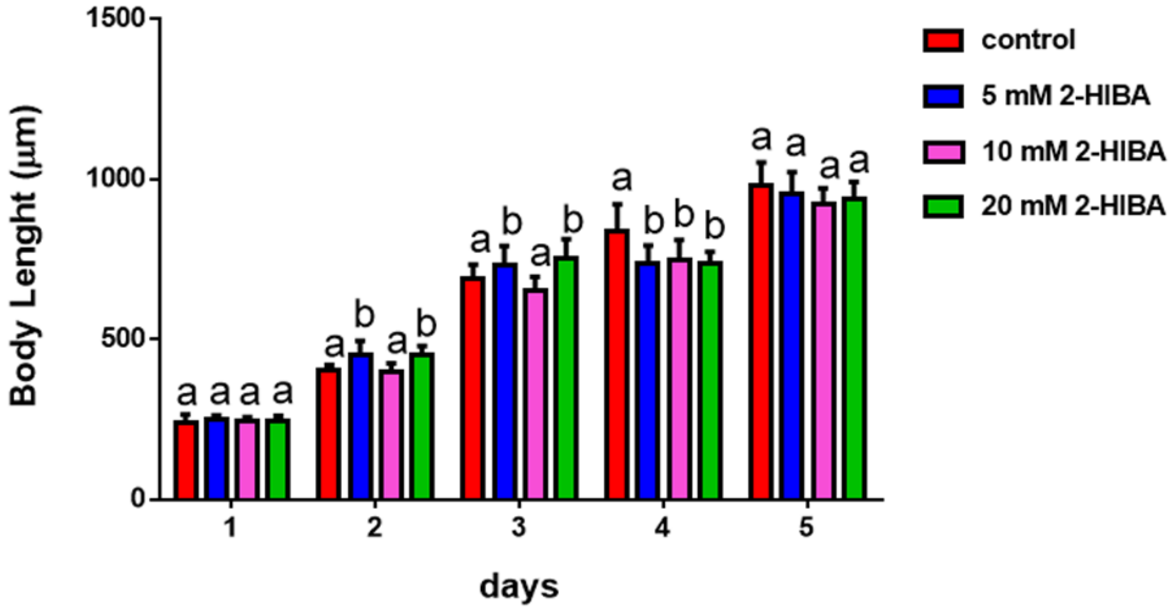

Figure S2. 600 MHz ^{1}H-NMR spectra of the *C. elegans* hydroalcoholic extracts in D_2O. Red spectrum: HGD worms supplemented with 2-HIBA 10 mM; black spectrum: untreated HGD worms. Regions: A) 0.5-2.5 ppm; B) 2.5-5.0 ppm; C) 5.0-9.5 ppm.

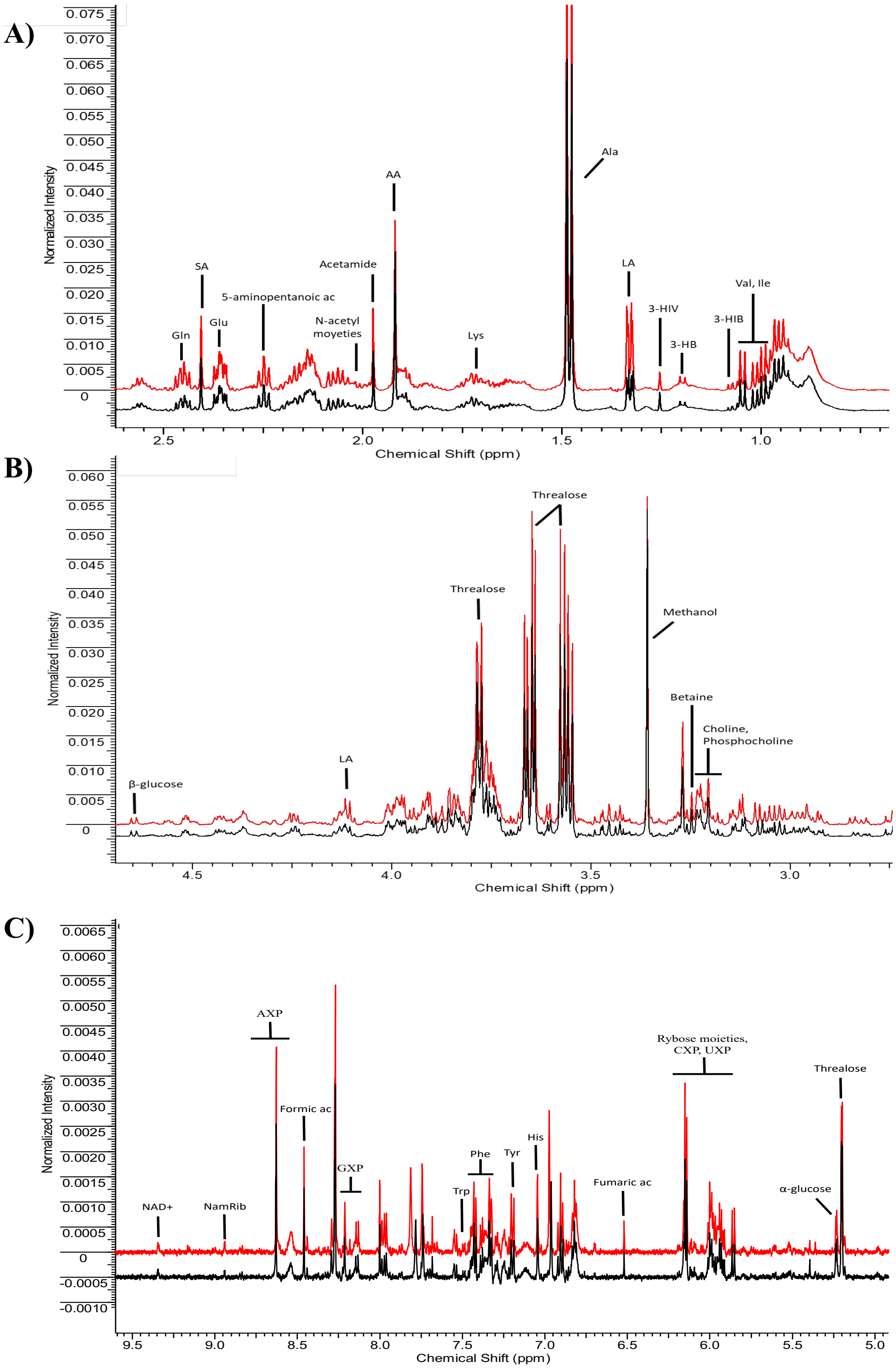

Figure S3. 600 MHz ^{1}H-NMR spectra of the C. elegans chloroformic extracts in $CDCl_3$. Red spectrum: HGD worms supplemented with 2-HIBA 10 mM; black spectrum: untreated HGD worms.

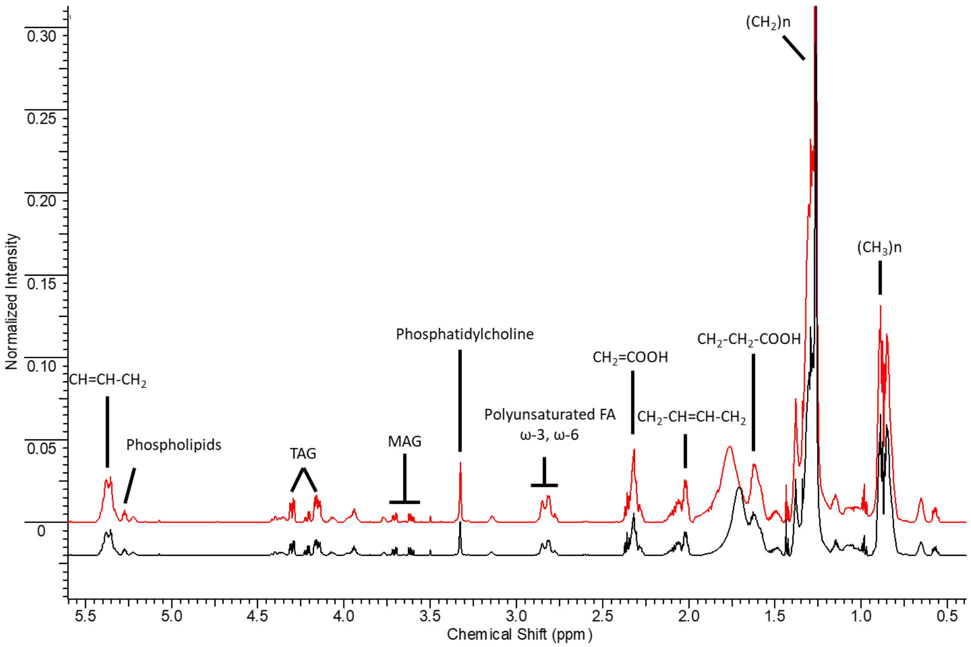

Figure S4. PCA analysis performed on the overall dataset of *C. elegans* samples. In red are shown the 14 samples grown with on a HGD and in blue the 14 samples grown without the addition of glucose in the substrate. The first principal component (PC1) accounted for 49% of the overall variance, while the second (PC2) accounted for 18%.

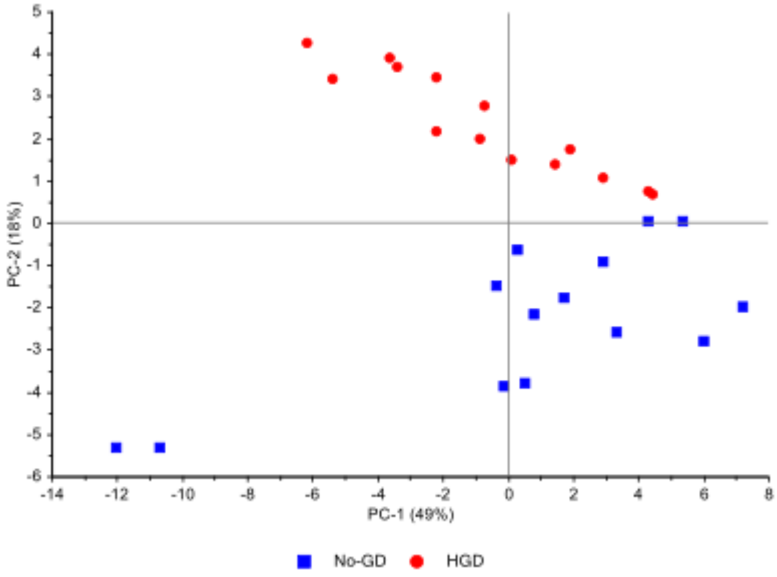

Supplement: Supplementary file 1 [file DataSheet1.PDF]
